# Supplementary material for: Dissected antiporter modules establish minimal proton-conduction elements of the respiratory complex I
Source: Nat Commun. 2024 Oct 22;15:9098. doi: 10.1038/s41467-024-53194-5 (PMC11496545; doi:10.1038/s41467-024-53194-5)
Supplement: Supplementary file 1 — Supplementary Information [file 41467_2024_53194_MOESM1_ESM.pdf]

Supplementary Information

Dissected Antiporter Modules Establish Minimal Proton-Conduction Elements of the Respiratory Complex I

Adel Beghiah[1], Patricia Saura[1,a], Sofia Badolato[1,a], Hyunho Kim[1], Johanna Zipf[1], Dirk Auman[1], Ana P. Gamiz-Hernandez[1], Johan Berg[1], Grant Kemp[1], Ville R. I. Kaila[1,*]

[1] Department of Biochemistry and Biophysics, Stockholm University, 10691, Stockholm, Sweden.
[a] These authors contributed equally.
[*]Corresponding author: Ville R. I. Kaila, **E-mail:** ville.kaila@dbb.su.se

Content

Supplementary Figure 1 | MD simulations of the dissected antiporter-like subunits.
Supplementary Figure 2 | Analysis of the proton channels in Nqo13 constructs.
Supplementary Figure 3 | Analysis of the proton channels in Nqo12$^{\Delta TH}$ constructs.
Supplementary Figure 4 | Effect of the membrane composition and protein-lipid interactions
Supplementary Figure 5 | Ion-pair dynamics in Nqo13 and Nqo12$^{\Delta TH}$ constructs.
Supplementary Figure 6 | Simulations details of the QM/MM calculations.
Supplementary Figure 7 | Analytical model geometry for electrostatic tuning effects.
Supplementary Figure 8 | Electric field effects along the proton transfer pathways.
Supplementary Figure 9 | Benchmarking the proton transfer energetics.
Supplementary Figure 10 | Construct design, expression, purification and stability of dissected antiporter-like modules.
Supplementary Figure 11 | Biophysical characterisation of proton conduction properties in dissected antiporter-like modules.
Supplementary Figure 12 | Calibration of pyranine fluorescence and data fitting.
Supplementary Figure 13 | Kinetic optimisation and characterisation of proton conduction in proteoliposomes.
Supplementary Figure 14 | Protonation dynamics in (proteo)liposomes reconstituted with ATP synthase.
Supplementary Figure 15 | Kinetic analysis of proton conduction in F_1F_o-Nqo proteoliposomes.
Supplementary Figure 16 | Analysis of pK_a tuning effects in the dissected antiporter-like subunit and the intact Complex I.
Supplementary Figure 17 | Formation of a Nqo12-Nqo13 complex.
Supplementary Figure 18 | Sequence conservation in Nqo12 and Nqo13.
Supplementary Figure 19 | Hydration analysis of the protein-lipid interface in the isolated antiporter constructs and in the full Complex I.
Supplementary Table 1 | Steady-state pH levels and kinetics of proton transfer
Supplementary Table 2 | Orientation of Nqo modules in proteoliposomes by NTA-Atto 647N labelling.
Supplementary Table 3 | Predicted pK_as of dissected antiporter-like subunit relative to the intact Complex I.
Supplementary Table 4 | List of MD simulations.
Supplementary Table 5 | List of designed primers.
Supplementary Table 6 | Sequences of studied Nqo constructs.
Supplementary Table 7 | Expression strains and tested plasmids.
Supplementary Table 8 | Benchmarking the proton transfer energetics.
Supplementary Table 9 | List of QM/MM simulations.

Supplementary Methods
Derivation of an analytical model for ion-pair induced barrier tuning
Poisson-Boltzmann Electrostatic with Monte Carlo sampling
Analysis of hydration profiles
Constant-pH MD simulations
Supplementary Discussion

Supplementary References

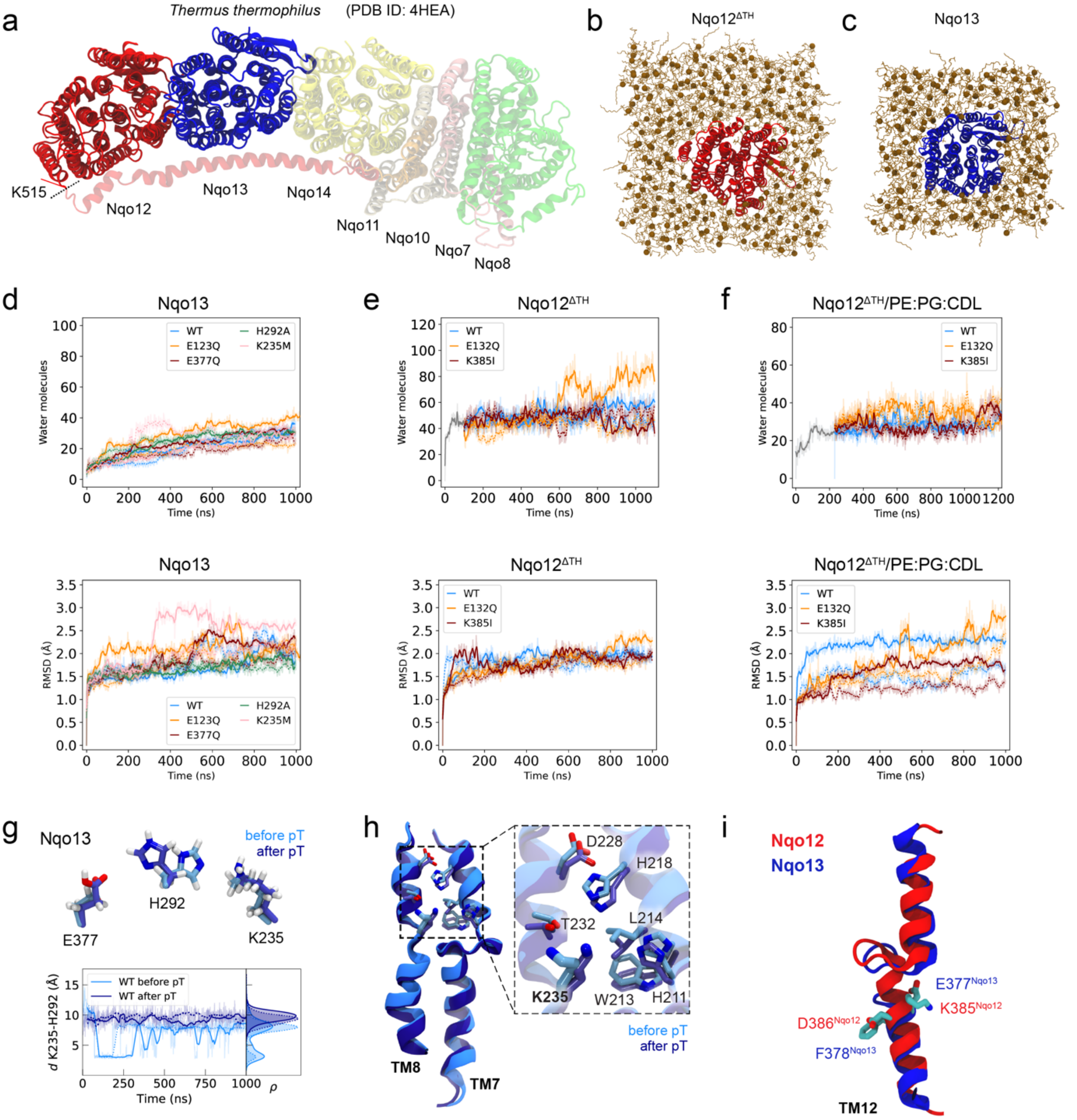

Supplementary Figure 1 | MD simulation of the dissected antiporter-like subunits. a) The coordinates of Nqo12 and Nqo13 were obtained from *T. thermophilus* Complex I (PDB ID: 4HEA). The transverse helix of Nqo12 was removed by truncating the protein at position 515 (Nqo12$^{\Delta TH}$), leaving Lys515 as the new C-terminus. **b**) Nqo12$^{\Delta TH}$ and **c**) Nqo13 embedded in a POPC lipid membrane (see *Methods*). **d-f**) RMSD (*bottom*) and number of water molecules within 5 Å of proton pathway (*top*) during the MD simulations of Nqo13 and Nqo12$^{\Delta TH}$ constructs. **e, f**) Probing the effect of the membrane composition for the different Nqo12$^{\Delta TH}$ constructs with MD simulations in **e**) POPC, and **f**) POPE:POPG:CDL membranes. **g**) *Top:* conformation of residues along the lateral proton pathway in Nqo13 (light blue: Lys235^{+}/His292^{0}/Glu377^{-}, dark blue: Lys235^{0}/His292^{0}/Glu377^{0}). *Bottom:* Distance between Lys235 (Nζ) and His292 (Nε) during MD simulations in Lys235^{+}/His292^{0}/Glu377^{-} (light blue) Lys235^{0}/His292^{0}/Glu377^{0} (dark blue) states. **h**) Conformational changes in conserved residues in TM7 and TM8 at the N-side proton pathway, upon proton transfer from Lys235 to Glu377 (light blue, before / dark blue, after proton transfer). **i**) Alignment of TM12 helix in Nqo12 (red) and Nqo13 (blue), showing the position of the terminal residues along the proton pathway (K385^{Nqo12}, E377^{Nqo13}) and the P-side exit (D386^{Nqo12}, F378^{Nqo13}). Data are provided in the Source Data file.

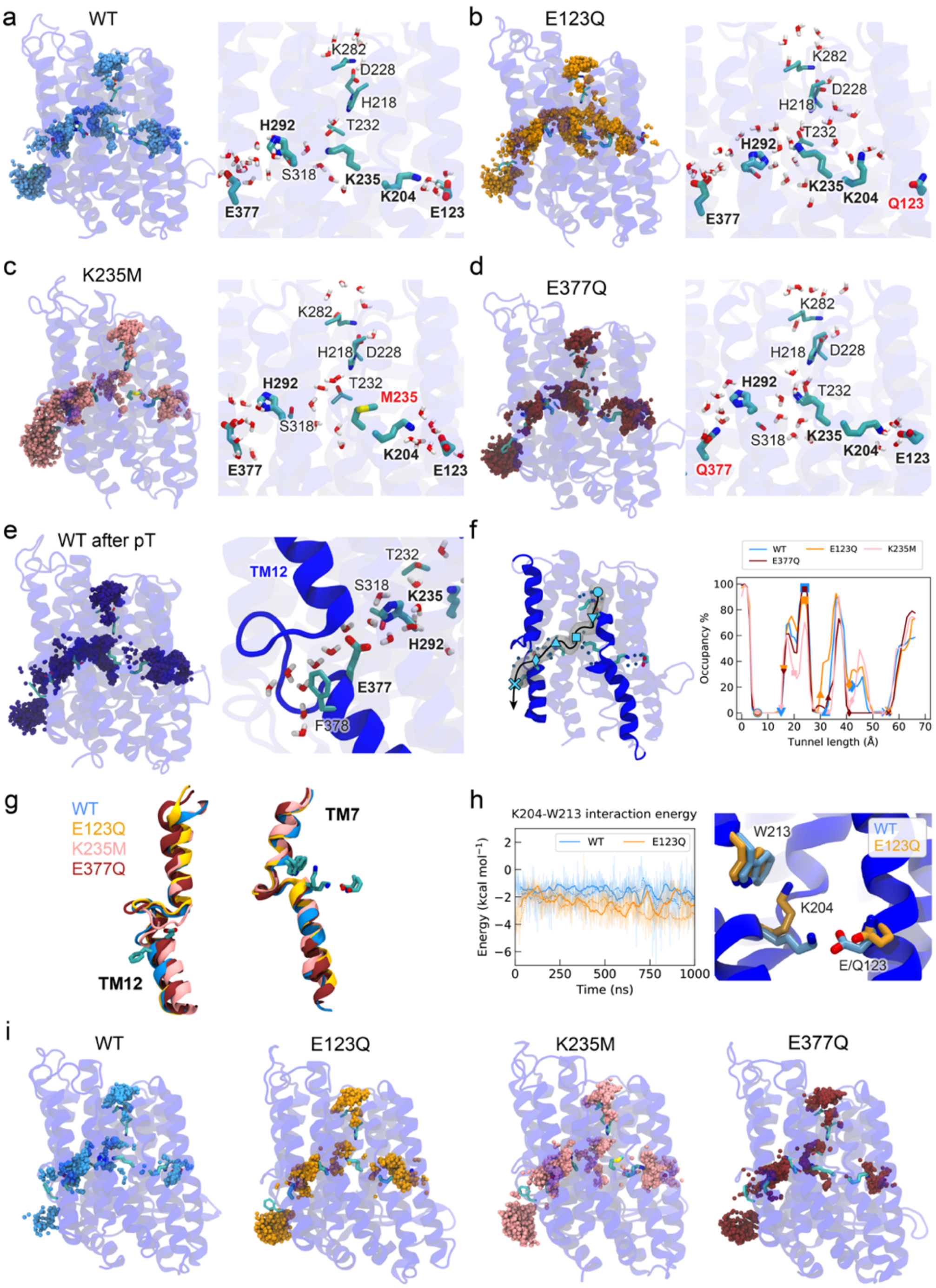

Supplementary Figure 2 | Analysis of the proton channels in Nqo13 constructs. a-f) *Left:* Hydration along the proton transfer pathway and ion-pair region, averaged over 1 µs MD simulations. *Right:* MD snapshot showing water connectivity and polar residues for **a**) WT before proton transfer from Lys235 to Glu377), **b**) E123Q, **c**) K235M, **d**) E377Q and **e)** WT after proton transfer from Lys235 to Glu377, with TM12 shown in solid colour (*inset, right*). **f**) Hydration level along the proton pathways for the different Nqo13 variants. Probe sites are indicated with symbols in the structure (*left*) and the hydration profile (*right*). **g**) Conformations of the transmembrane broken helices TM12 and TM7 in the different Nqo13 constructs. **h)** The conserved Trp213 stabilises Lys204 by π-cation interactions in the open ion-pair conformation. The figure shows the interaction energy between Lys204-Trp213 (*left*) and structures from MD simulations (from independent replicas) of WT and E123Q (*right*). **i**) Hydration along the proton pathways in the Nqo13 constructs (WT, E123Q, K235M and E377Q). The data is averaged over 0.5 µs MD simulations in a PE:PG:CDL membrane (see Methods). Data are provided in the Source Data file.

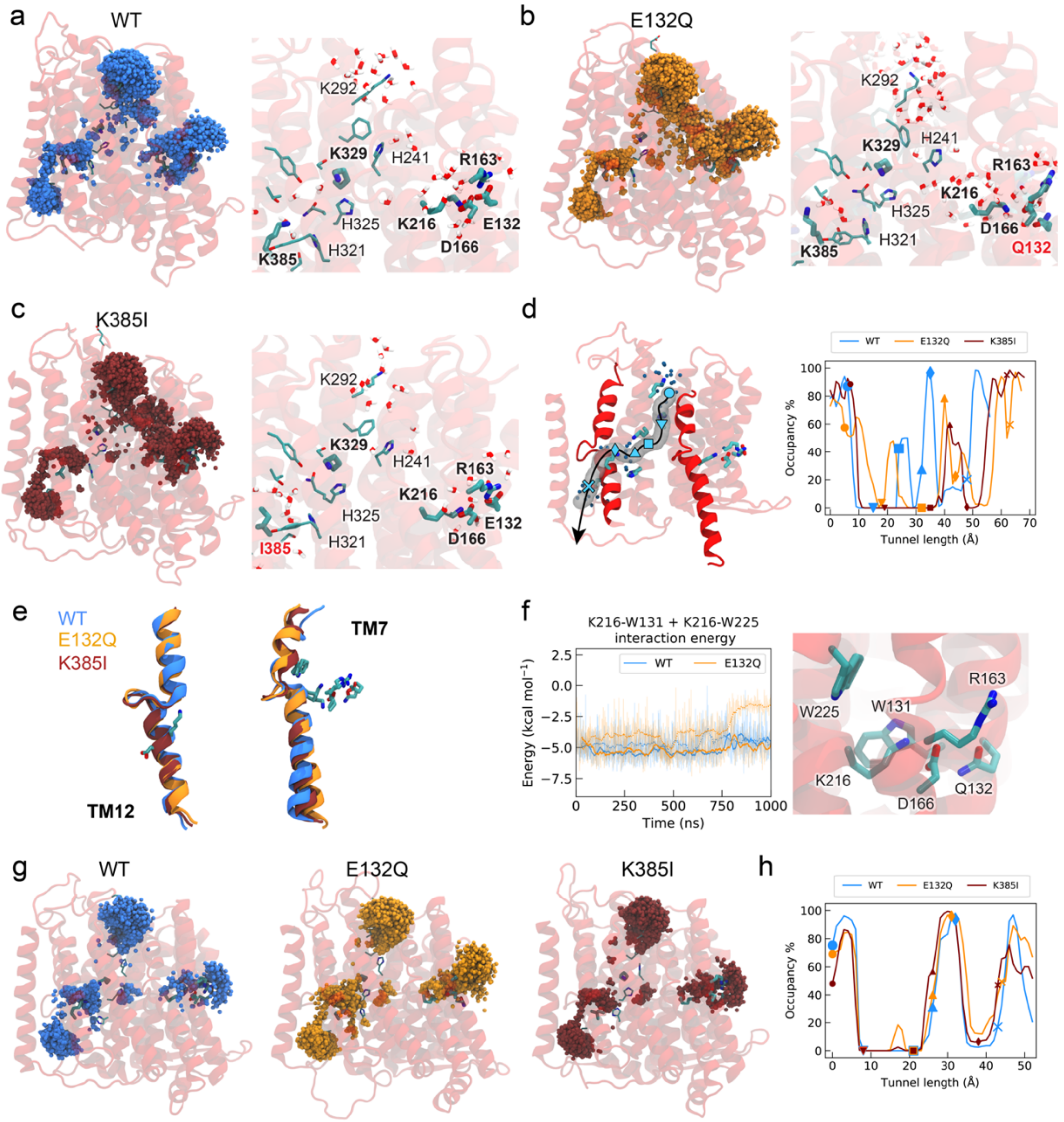

Supplementary Figure 3 | Analysis of the proton channels in Nqo12$^{\Delta TH}$ constructs. a-c) *Left:* Hydration along the proton transfer pathway and ion-pair region, averaged over 1 µs simulations in a POPC membrane. *Right:* MD snapshot showing water connectivity and polar residues in **a**) WT, **b**) E132Q, and **c**) K385I. **d**) Hydration level along the proton pathways (*left*) for the different Nqo12$^{\Delta TH}$ constructs. **e**) Conformation of the transmembrane broken helices TM12 and TM7 in the different Nqo12$^{\Delta TH}$ constructs. **f**) Trp225/Trp131-K216 interaction in WT and ion-pair mutant. **g**) Hydration along the proton pathways in the Nqo12$^{\Delta TH}$ constructs (WT, E132Q, and K385I). The data is averaged over 1 µs MD simulations in a PE:PG:CDL membrane (see *Methods*). **h**) Hydration profile along the proton pathway in the Nqo12$^{\Delta TH}$ constructs. Data are provided in the Source Data file.

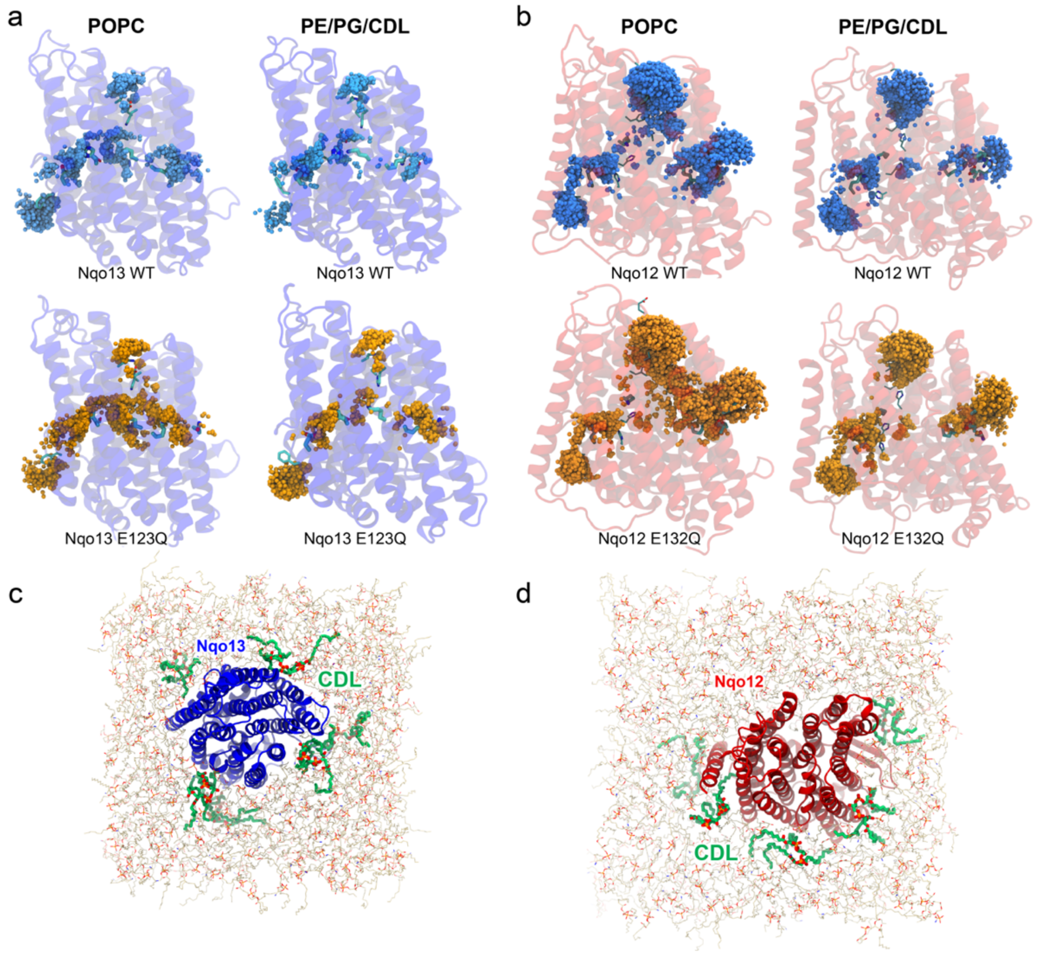

Supplementary Figure 4 | Effect of the membrane composition and protein-lipid interactions. a, b) Hydration along the proton pathways in (**a**) Nqo13 and (**b**) Nqo12$^{\Delta TH}$ constructs in a pure POPC membrane and in a PE:PG:CDL mixture for the WT (water molecules in blue) and ion-pair mutant (water molecules in orange) simulations. **c, d**) Cardiolipin (CDL) molecules stabilise the protein-lipid interface in the MD simulations with the PE:PG:CDL membrane for **c**, Nqo13 and **d**, Nqo12$^{\Delta TH}$.

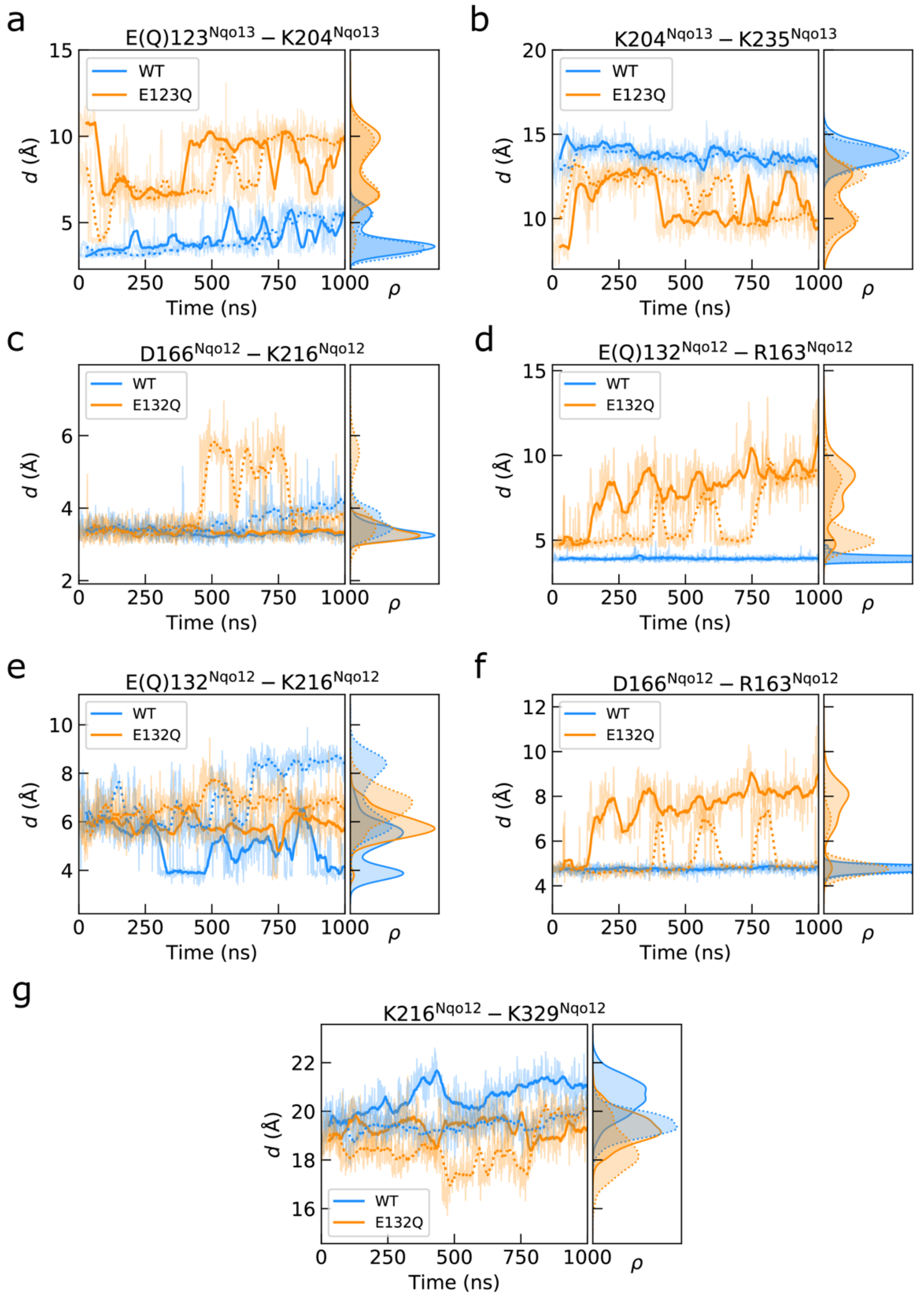

Supplementary Figure 5 | Ion-pair dynamics in Nqo13 and Nqo12$^{\Delta TH}$ constructs. Blue traces correspond to WT, and orange traces to ion-pair mutant constructs. Distances between **a**) E/Q123-K204 and **b**) K204-K235 in Nqo13. Distances between **c**) D166/K216, **d**) E/Q132-R163, **e**) E/Q132-K216, **f**) D166-R163), and **g**) K216-K329 in Nqo12$^{\Delta TH}$. Data are provided in the Source Data file.

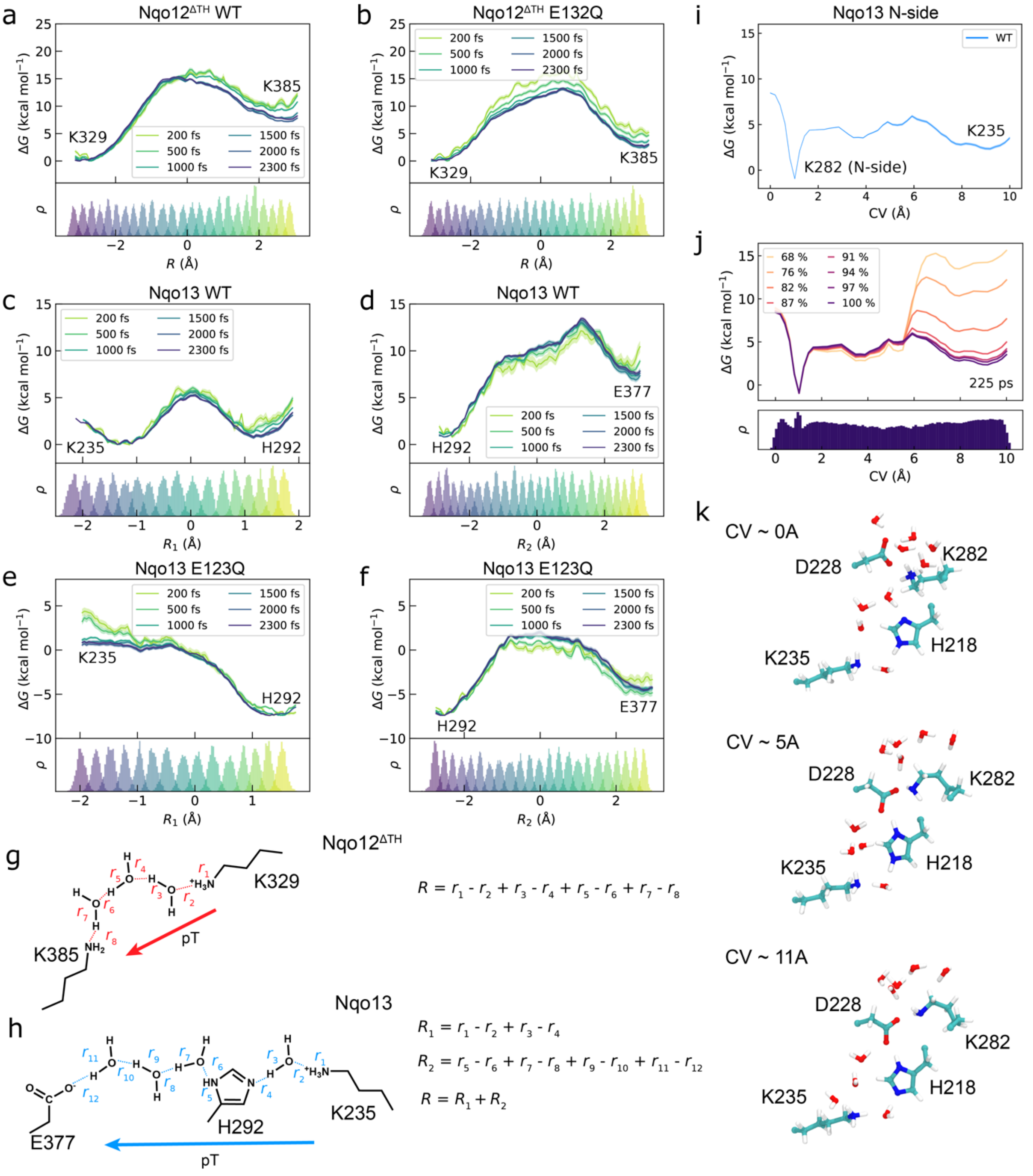

$R = r_1 - r_2 + r_3 - r_4 + r_5 - r_6 + r_7 - r_8$

$R_1 = r_1 - r_2 + r_3 - r_4$

$R_2 = r_5 - r_6 + r_7 - r_8 + r_9 - r_{10} + r_{11} - r_{12}$

$R = R_1 + R_2$

Supplementary Figure 6 | Simulation details of the QM/MM calculations. a-f) Convergence of free energy profiles (top) and overlap of the umbrella sampling windows (*bottom*) for the lateral proton transfer reactions in the different systems. Proton transfer from K329 to K385 in **a)** WT and **b)** E132Q variant of Nqo12$^{\Delta TH}$. Proton transfer from **c)** K235 to H292 and **d)** H292 to E377 in WT-Nqo13. Proton transfer from **e)** K235 to H292 and **f)** H292 to E377 in E123Q -Nqo13. **g-h)** The reaction coordinate (R) for the lateral proton transfer reaction in **g)** Nqo12$^{\Delta TH}$ and **h)** Nqo13, defined as a linear combination of bond-breaking and bond-forming distances. **i)** The free energy of proton uptake from the N-side to the middle Lys235 in Nqo13, using the modified centre of excess charge (mCEC) as a reaction coordinate / collective variable (CV (Å), see *Methods*). **j)** Convergence of the MWE-QM/MM free energy profiles with increasing sampling time. **k)** Snapshots along the N-side proton transfer reaction at different CVs, from the N-side (CV = 0 Å) to the middle Lys235 (CV = 11 Å). Data are provided in the Source Data file.

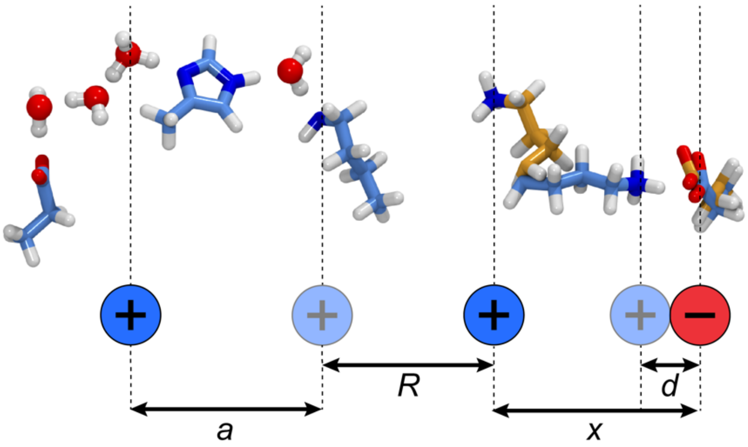

Supplementary Figure 7 | Analytical model geometry for electrostatic tuning effects. *R* - distance between 'middle Lys' and 'ion-pair Lys'; *d* - distance between ion-pairs in closed conformation; *x* - distance between ion-pairs in the open conformation; *a* - position of transition state relative to the 'middle Lys'.

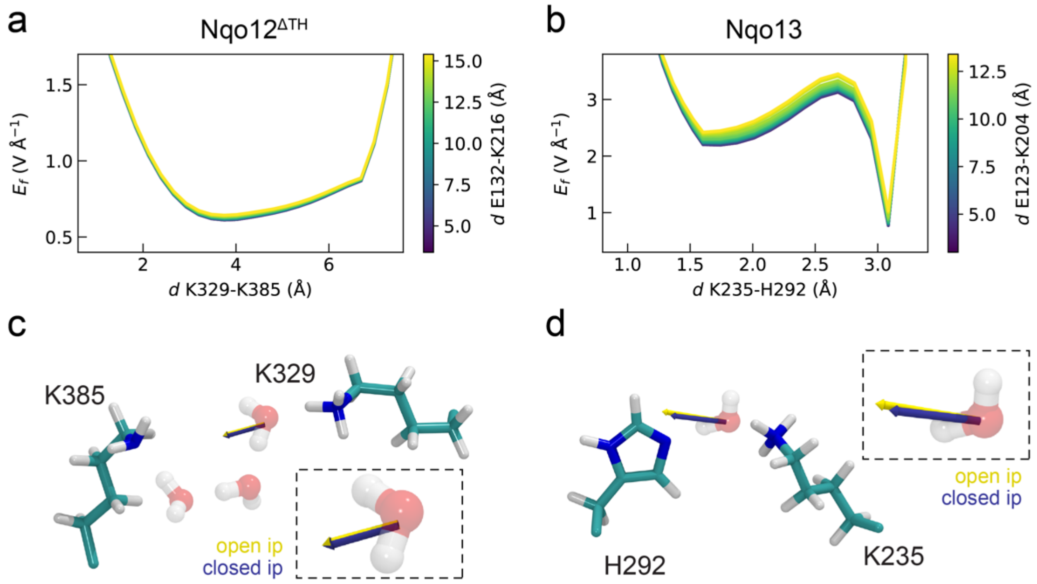

Supplementary Figure 8 | Electric field effects along the proton transfer pathways. Opening of the ion-pair induces an electric field along the proton transfer pathway in QM/MM calculations. **a**) Electric field strength along the proton transfer pathway from K329 to K385, with varying K216-E132 ion-pair distances (closed conformation in blue, open conformation in yellow) for Nqo12. **b**) Electric field strength along the proton transfer pathway from K235 to H292, with varying K204-E123 ion-pair distances (closed conformation in blue, open conformation in yellow) for Nqo13. **c-d**) Electric field vectors (closed ion-pair (blue)/open ion-pair (yellow)) for proton pathways in **c**) Nqo12 and **d**) Nqo13. Insets show a closeup of the electric field vectors. Data are provided in the Source Data file.

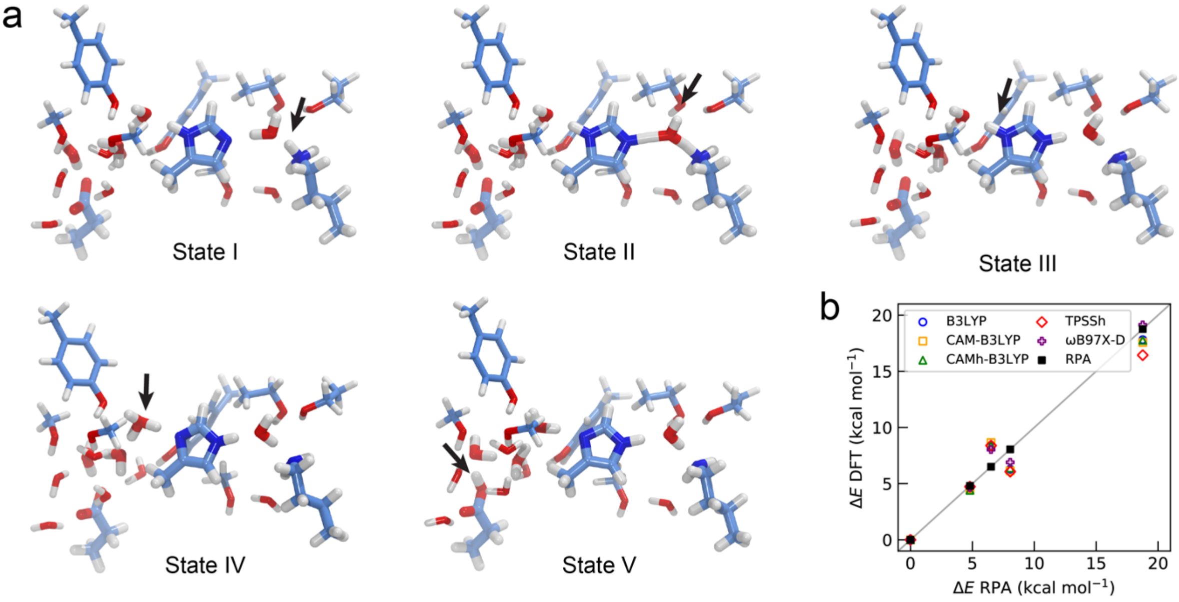

Supplementary Figure 9 | Benchmarking the proton transfer energetics. a) QM model systems, comprising 137 atoms, employed for the benchmarking calculations of the proton transfer reactions. The position of the proton in the different states (states I - V) is indicated by an arrow. **b**) DFT electronic energies with different functionals vs. RPA energies. See also Supplementary Table 8. Data are provided in the Source Data file.

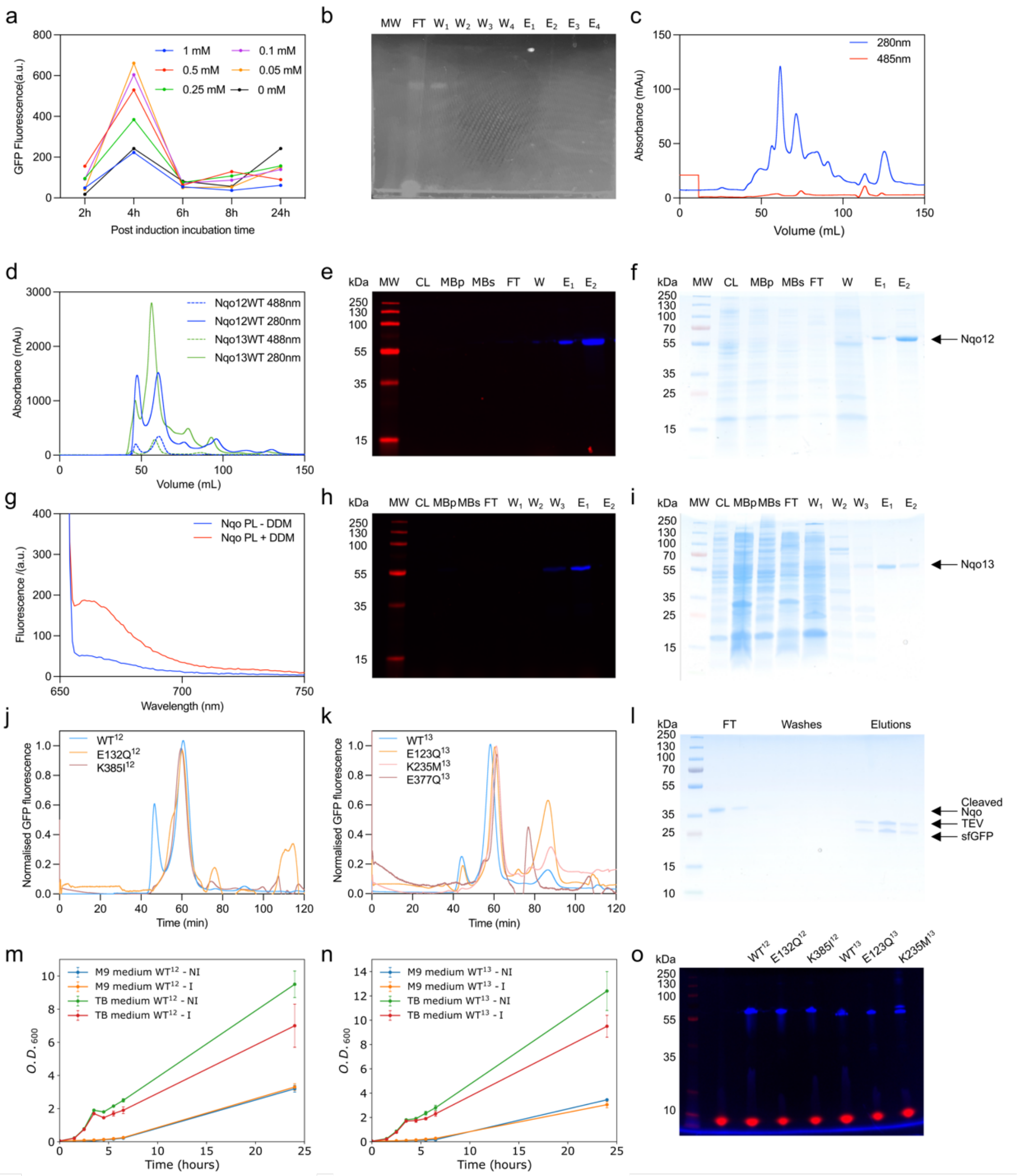

Supplementary Figure 10 | Construct design, expression, purification, and stability of dissected antiporter-like modules. **a**) Nqo12$^{\Delta TH}$ expression trial using BL21 strain followed by GFP in-cell fluorescence. **b**) In-gel fluorescence of Nqo12$^{\Delta TH}$-sfGFP (without linker) monitoring IMAC fractions. **c**) Size-exclusion chromatography profile showing the absorbance at 280 nm and GFP excitation at 488 nm of Nqo12$^{\Delta TH}$-sfGFP without linker expressed in BL21. **d**) Size-exclusion chromatography profiles showing the absorbance at 280 nm and GFP excitation at 488 nm. **e**) In-gel fluorescence and **f**) Coomassie gel of Nqo12$^{\Delta TH}$ following the purification fractions (CL: cell lysate, MBp: membranes pellet, MBs: membrane centrifugation supernatant, FT: flow through, W: wash, E_1: IMAC elution, E_2: SEC elution). **g**) Nqo orientation in liposomes measured using the NTA–Atto 647 N fluorescent label. **h**) In-gel fluorescence image and **i**) Coomassie gel of Nqo13 following the purification fractions (CL: cell lysate, MBp: membranes pellet, MBs: membrane centrifugation supernatant, FT: flow through, $W_{1\text{-}2\text{-}3}$: washes, E_1: IMAC elution, E_2: SEC elution). SEC profiles for **j**) Nqo12 and **k**) Nqo13, eluted at 60 mL, showing GFP fluorescence followed at 488 nm. **l**) Gel showing sfGFP removal by overnight cleavage with TEV protease. Growth tests performed in TB and minimum medium (M9) for **m**) Nqo12 and **n**) Nqo13 in induced (I) and non-induced (NI) conditions. **o**) In-gel fluorescence assessing the reconstitution efficiency of the Nqos into proteoliposomes. Data are provided in the Source Data file.

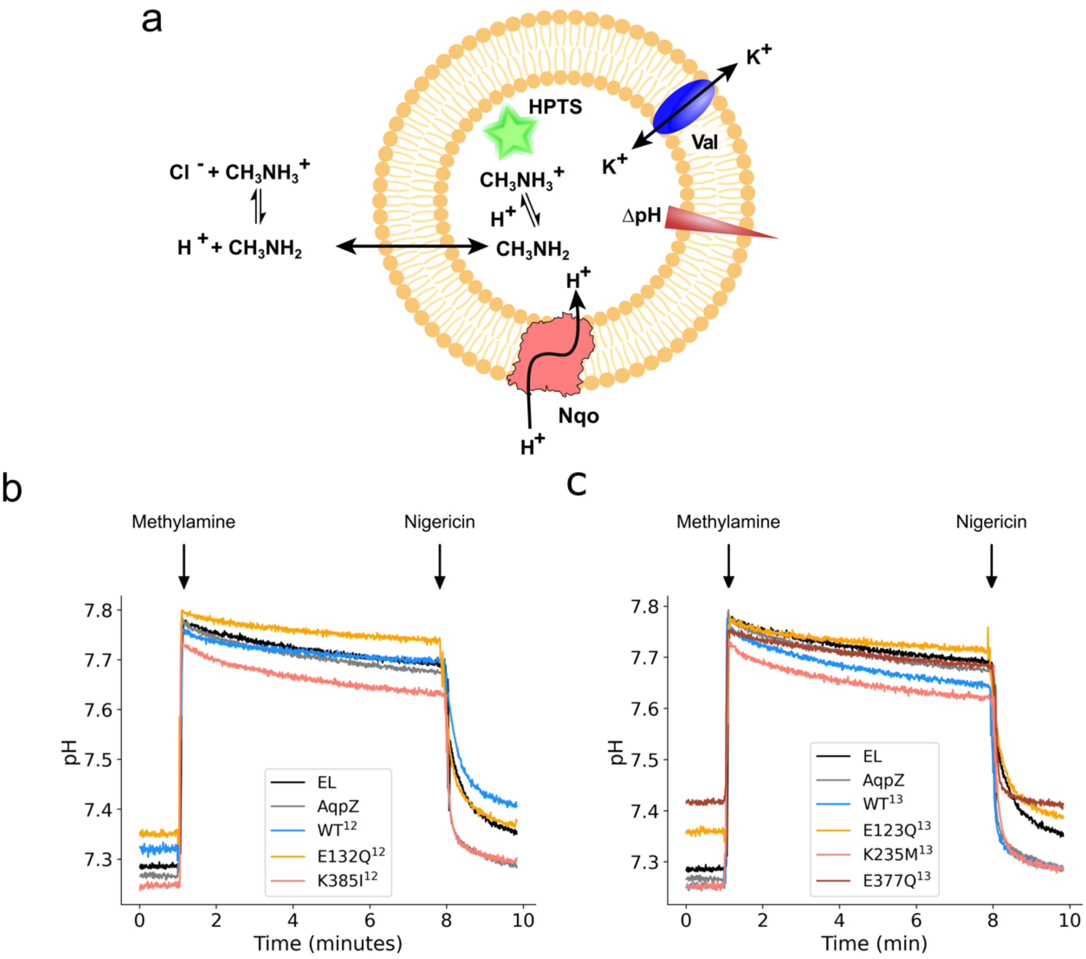

Supplementary Figure 11 | Biophysical characterisation of proton conduction properties in dissected antiporter-like modules. a) Proteoliposome assays for probing the proton conduction kinetics in the dissected antiporter-like subunits with pyranine (HPTS) by addition of $CH_3NH_3^+Cl^-$. Proton conduction in **b**) Nqo12 and **c**) Nqo13 proteoliposomes upon addition of 10 mM methylamine hydrochloride. Methylamine (pK_a=10.6) addition leads to smaller kinetic differences between the constructs as compared to the acetate (pK_a=4.8) assays, possibly due to the shifted equilibrium between the acid and base forms ([acid]/[base] = $10^{3.4}$ amine vs. $10^{2.4}$ acetate at pH 7.2), secondary reactions between hydroxide ions and $CH_3NH_3^+$, and/or differences in membrane permeability of the weak acid/base ($P_{CH3COOH}$ = 6.9 x 10^{-3} cm s^{-1}, P_{CH3NH2} = 9 x 10^{-1} - 8 x 10^{-2} cm s^{-1}). Data are provided in the Source Data file.

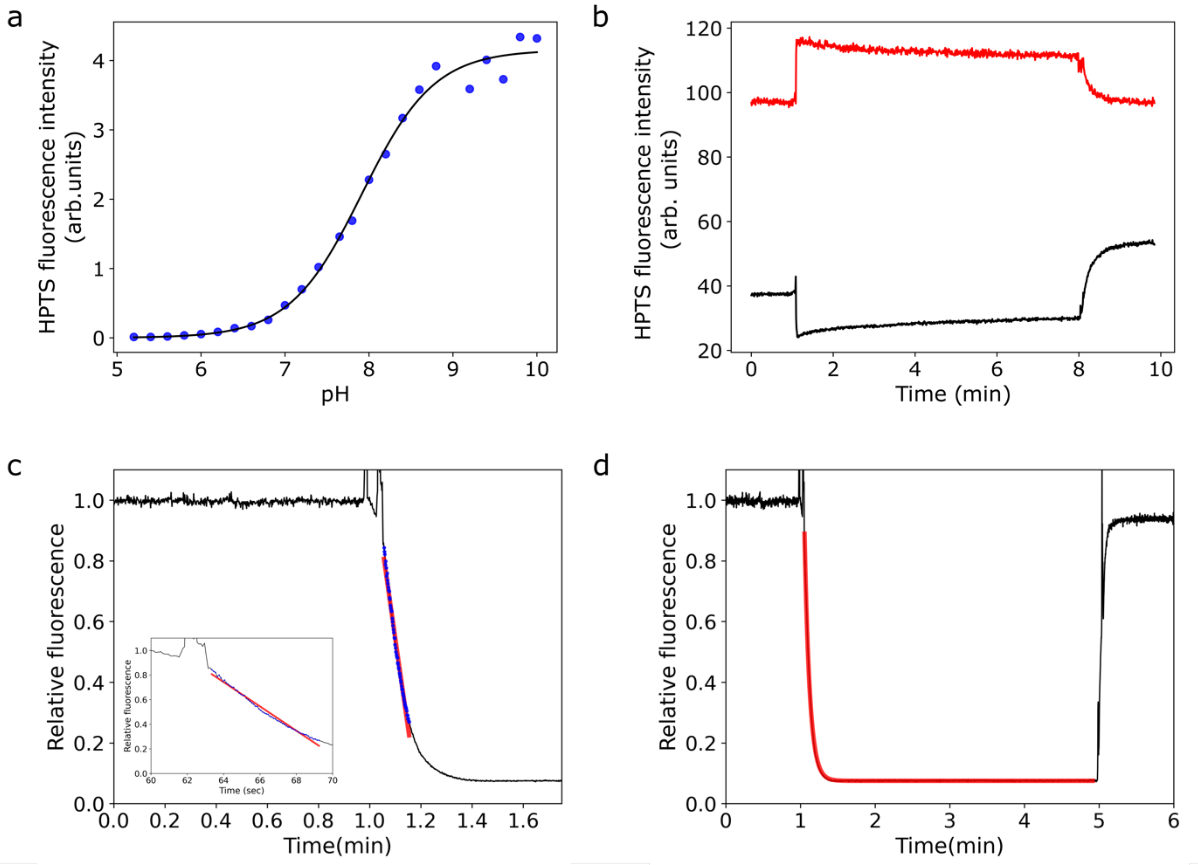

Supplementary Figure 12 | Calibration of pyranine fluorescence and data fitting. a) Pyranine calibration curve measured between pH 5 and 10. The higher measuring temperature (37°C) as well as the buffer conditions lead to an upshift in the pyranine pK_a from 7.2 (*cf. e.g.* Ref[15] for exploration of buffer effects). **b**) Raw data for pyranine fluorescence measurements following excitation at λ_{ex}=404 nm (*red*) and λ_{ex}=454 nm (*black*) and emission at λ_{em}=510 nm. ATP synthase data fitted using **c**) a linear regression or **d**) an exponential fit. Data are provided in the Source Data file.

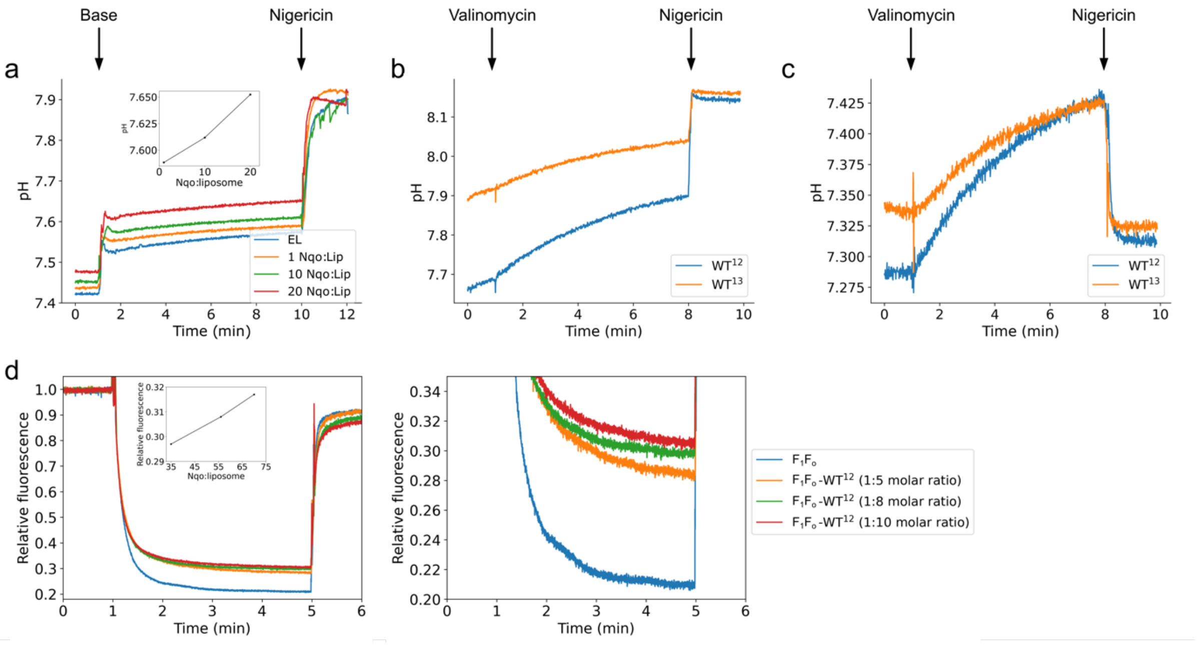

Supplementary Figure 13 | Kinetic optimisation and characterisation of proton conduction in proteoliposomes. a) Proton conduction monitored in the dissected antiporter-like subunits reconstituted in proteoliposomes with different Nqo-per-liposome ratio. The *pmf* was generated by addition of NaOH. *Inset*: linear relationship between the Nqo:liposome ratio and the resulting steady-state pH level upon addition of base. **b, c)** WT-Nqo12 and WT-Nqo13 mediated proton conduction induced by an external **b**) ΔpH- or **c**) ΔΨ- gradient, followed by addition of valinomycin (at 1 min). **d**) *Left*: ACMA fluorescence quench upon addition of ATP with different Nqo:F_1F_o ratios. *Inset*: linear relationship between the Nqo:liposome ratio and the resulting steady-state pH level upon addition of ATP. *Right*: Closeup of the quenching levels. Data are provided in the Source Data file.

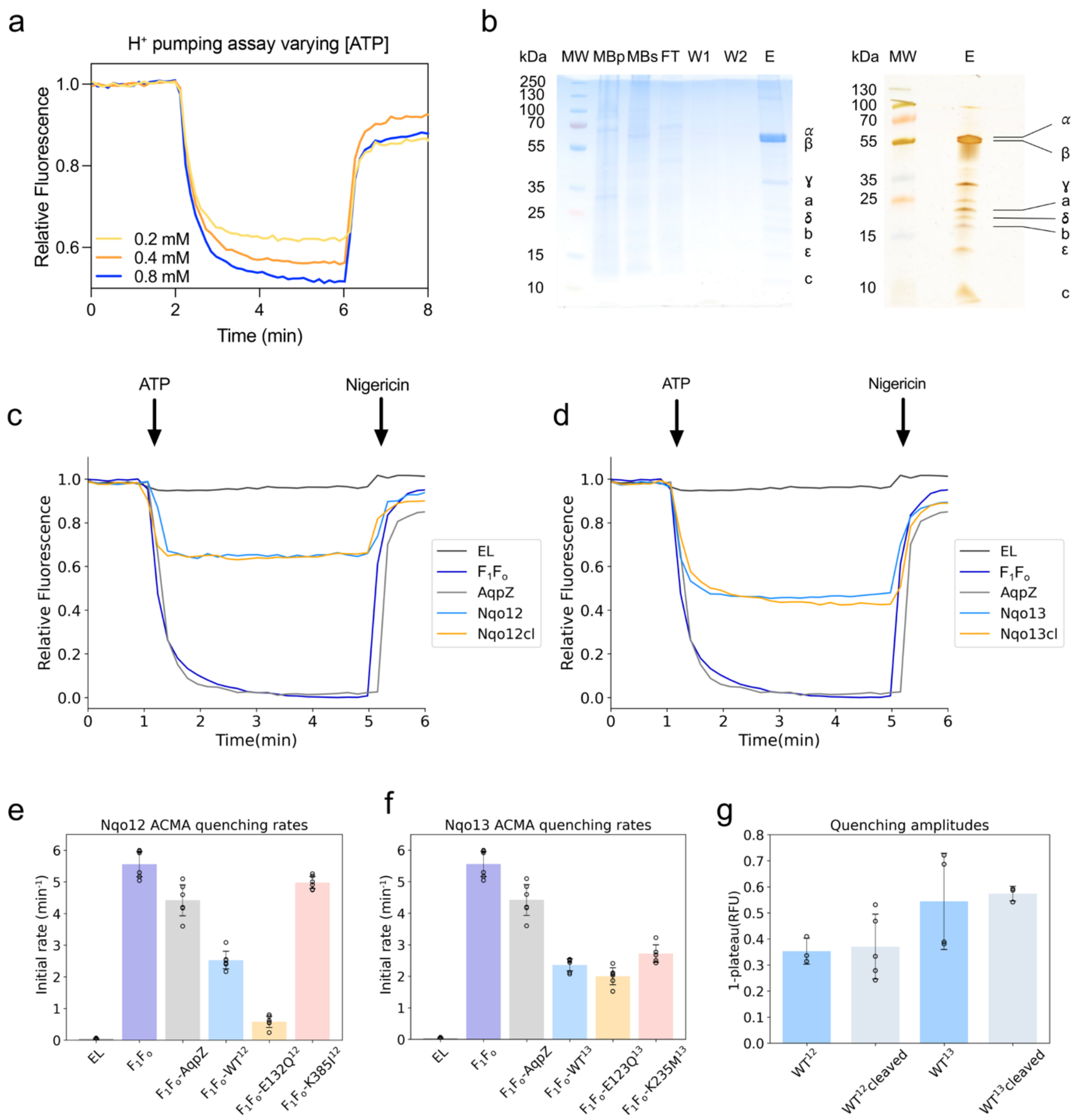

Supplementary Figure 14 | Protonation dynamics in (proteo)liposomes reconstituted with ATP synthase. **a**) Effect of ATP concentration on the ACMA fluorescence quenching. **b**) Coomassie gel of ATP synthase following the purification fractions (MBp: membranes pellet, MBs: membranes supernatant, FT: flow through, W1-2: washes, E: elution, and silver strained ATP synthase elution fraction. Proton conduction of **c**) Nqo12 and **d**) Nqo13 co-reconstituted with ATP synthase followed by ACMA fluorescence quenching. cl - sfGFP cleaved constructs. **e, f**) Fitted ACMA quenching rates from proteoliposome experiments with **e**) ATP synthase-Nqo12 and **f**) ATP synthase-Nqo13. Data shown are derived from independent experiments where n = 6 (mean ± SD) except F_1F_o-K235M[13] where n=5. **g**) Effect of cleaving sfGFP on the ACMA quenching for Nqo12 (n_{WT12}=4, $n_{WT12\ cleaved}$=3) and Nqo13 (n_{WT13}=3, $n_{WT13\ cleaved}$=5). Data are provided in the Source Data file.

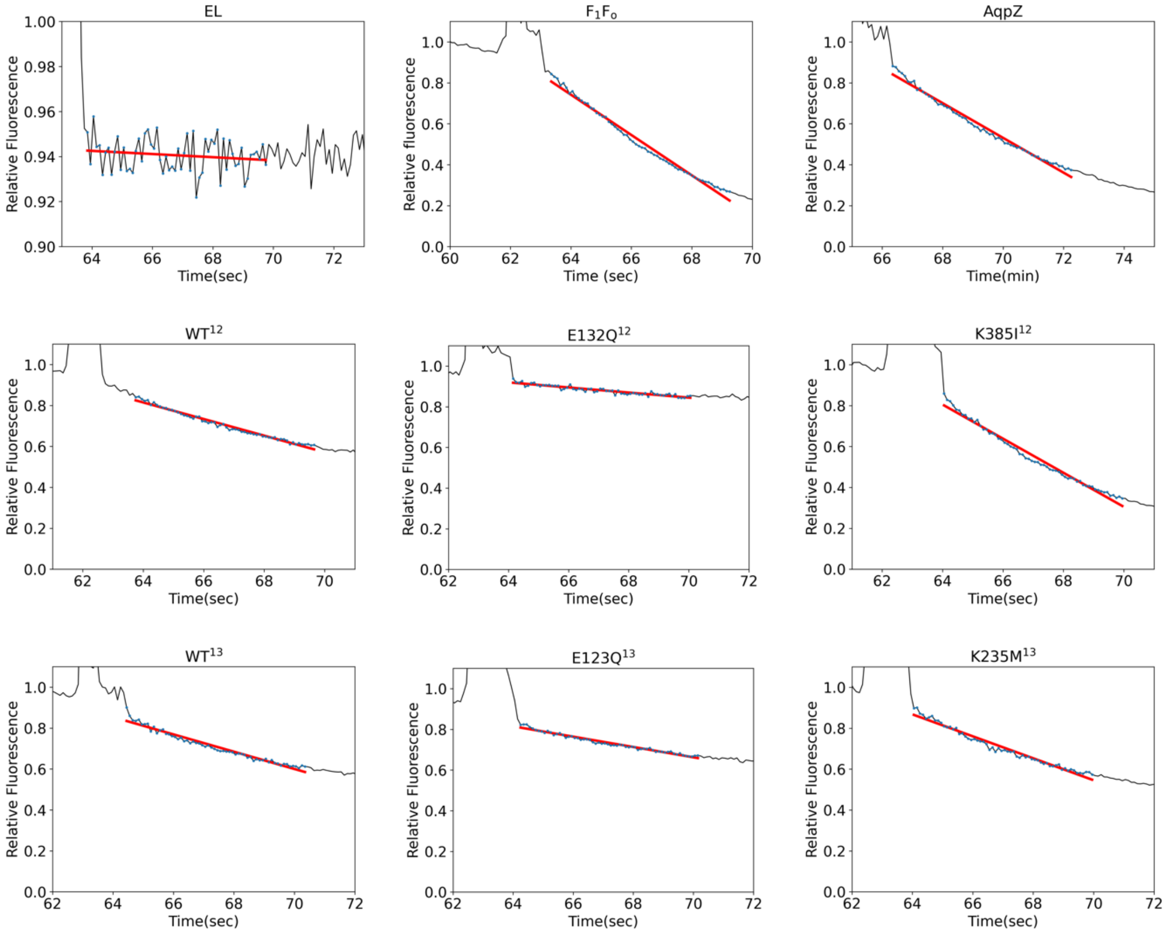

Supplementary Figure 15 | Kinetic analysis of proton conduction in F_1F_o-Nqo proteoliposomes. Proton conduction monitored in the dissected antiporter-like subunits reconstituted in proteoliposomes, with the *pmf* generated by addition of ATP with co-reconstituted ATP synthase using a linear regression fit. Data are provided in the Source Data file.

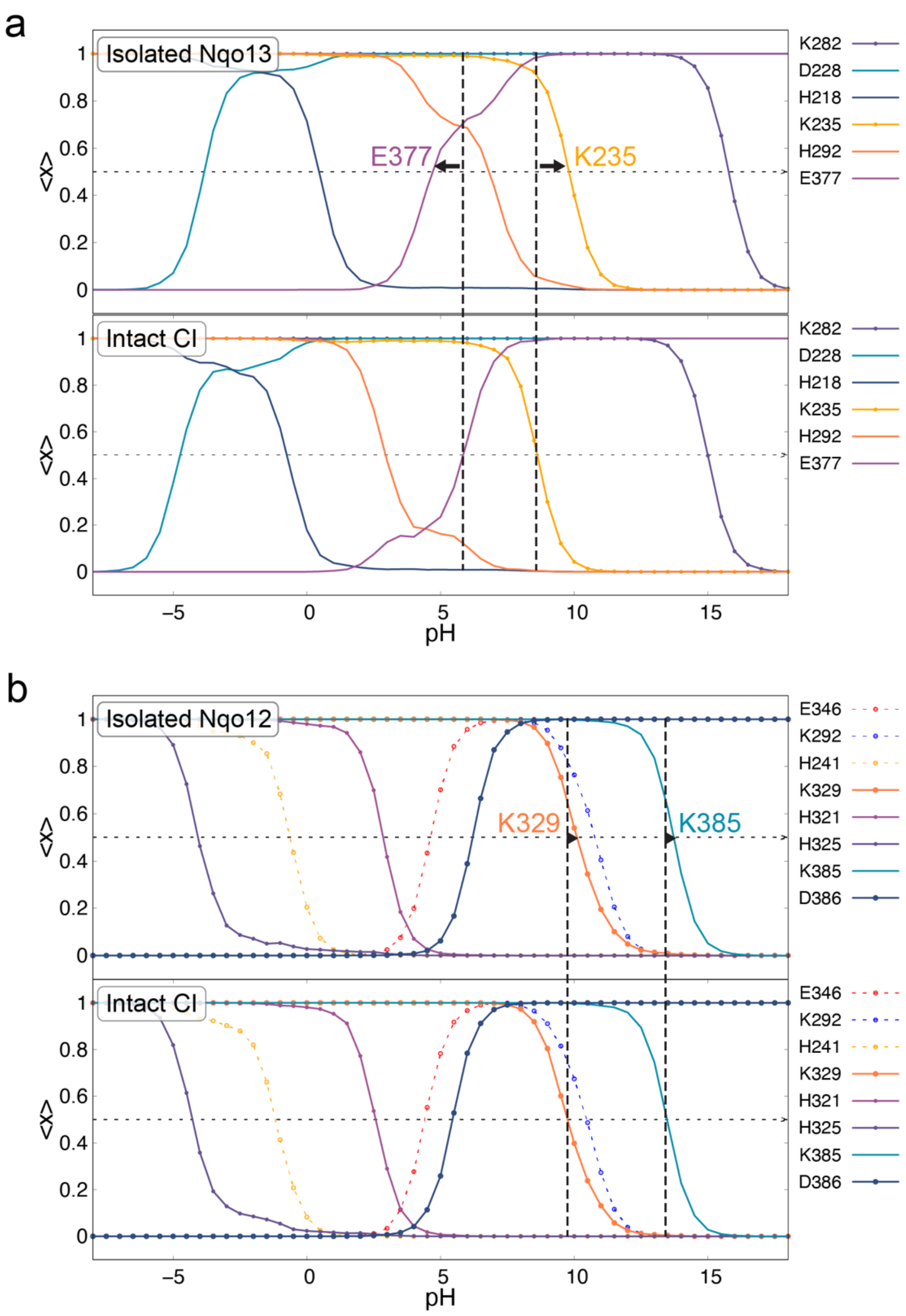

Supplementary Figure 16 | Analysis of pK_a tuning effects in the dissected antiporter-like subunit and the intact Complex I. The figure shows the protonation probability (<x>) as a function of pH. **a**) Titration curves of residues along the proton pathway for the isolated Nqo13 subunit (*top*), and Nqo13 within the intact Complex I (*bottom*), and **b**) the isolated Nqo12 subunit (*top*), and Nqo12 within the intact Complex I (*bottom*). Data are provided in the Source Data file.

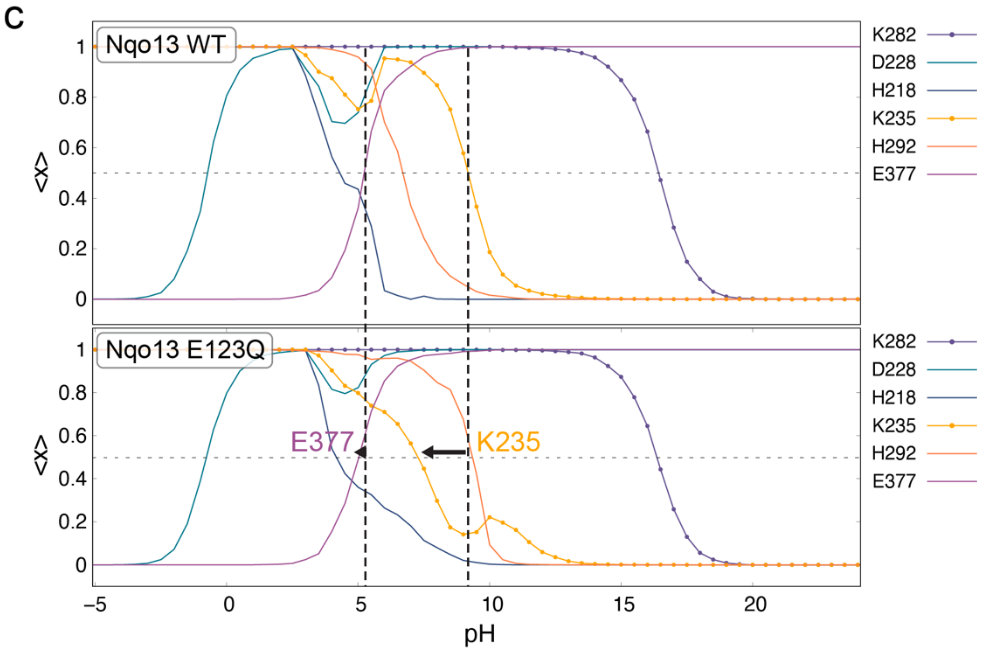

Supplementary Figure 16 (contd.) | Analysis of pK_a tuning effects in the dissected antiporter-like subunit and the intact Complex I. The figure shows the protonation probability (<x>) as a function of pH. **c**) Titration curves of residues along the proton pathway for the Nqo13 WT (*top*), and Nqo13 E123Q constructs, based on constant-pH MD simulations (see *Extended Methods*). Data are provided in the Source Data file.

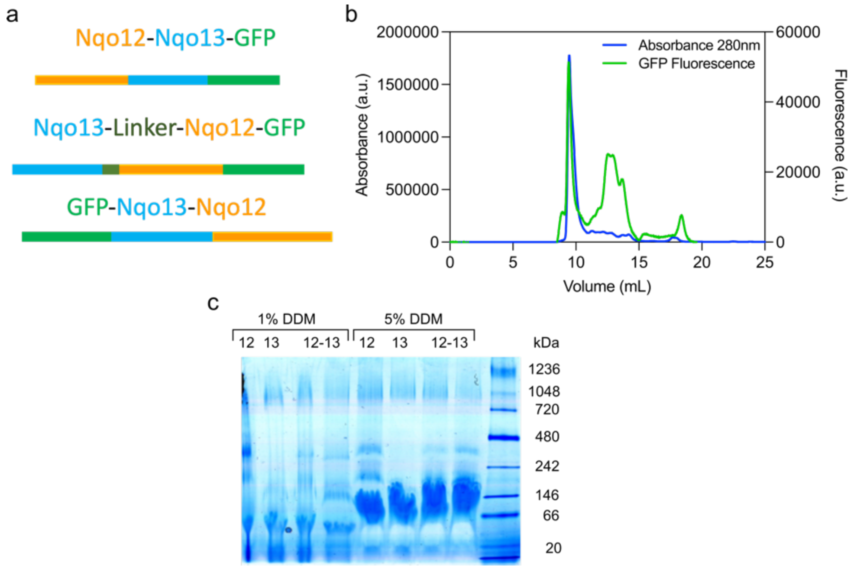

Supplementary Figure 17 | Formation of a Nqo12-Nqo13 complex. a) Design of an Nqo12-Nqo13 complex constructs. **b**) FSEC profile of truncated Nqo12 (ΔA571-T583)-Nqo13-GFP complex membranes with the protein content followed at 280 nm (blue) and GFP fluorescence followed at 485 nm (green). **c**) Blue-native PAGE of Nqo12$^{\Delta TH}$ and Nqo13 samples reconstituted separately and co-reconstituted in liposomes with *E. coli* polar lipids, followed by solubilisation in 1% or 5% DDM. The putative Nqo12$^{\Delta TH}$-13 complex was reconstituted using a protein to lipid ratio of 1:70 on the left and 1:140 on the right. Data are provided in the Source Data file.

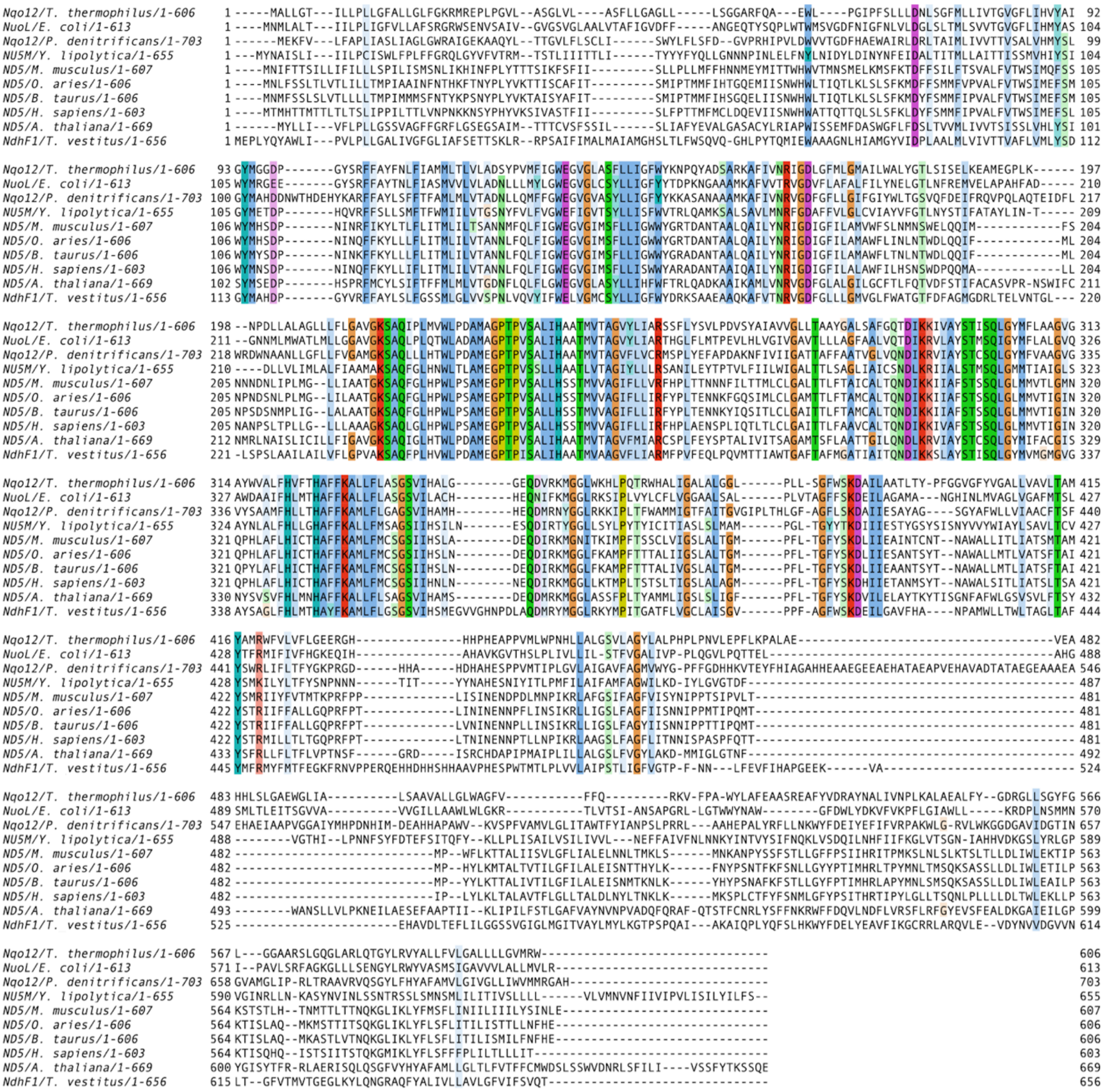

Supplementary Figure 18 | Sequence conservation in Nqo12 and Nqo13. Multiple sequence alignment (MSA) of Nqo12 and Nqo13 from different species. The MSA was performed with ClustalΩ.[16] Residues with up to 50% sequence conservation are coloured by the ClustalX colouring scheme.

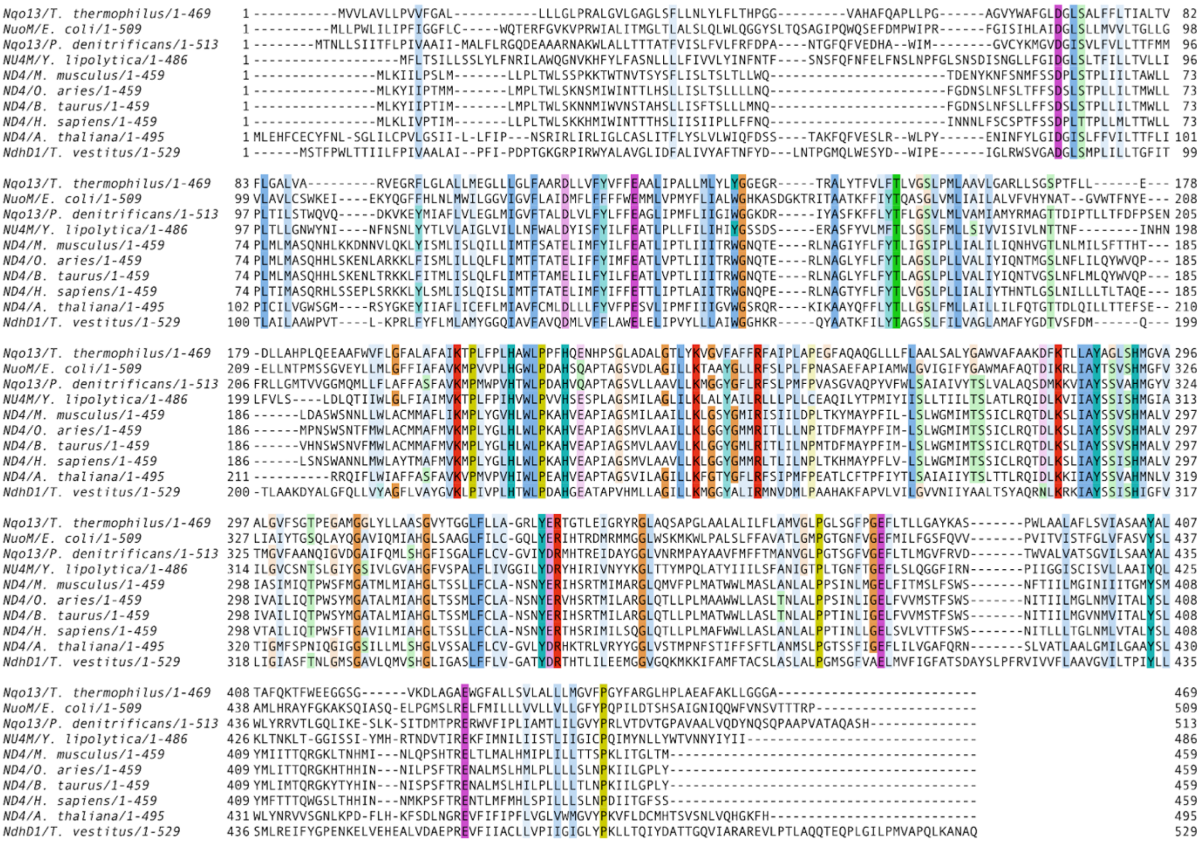

Supplementary Figure 18 (contd) | Sequence conservation in Nqo12 and Nqo13. Multiple sequence alignment (MSA) of Nqo12 and Nqo13 from different species. The MSA was performed with ClustalΩ.[16] Residues with up to 50% sequence conservation are coloured by the ClustalX colouring scheme.

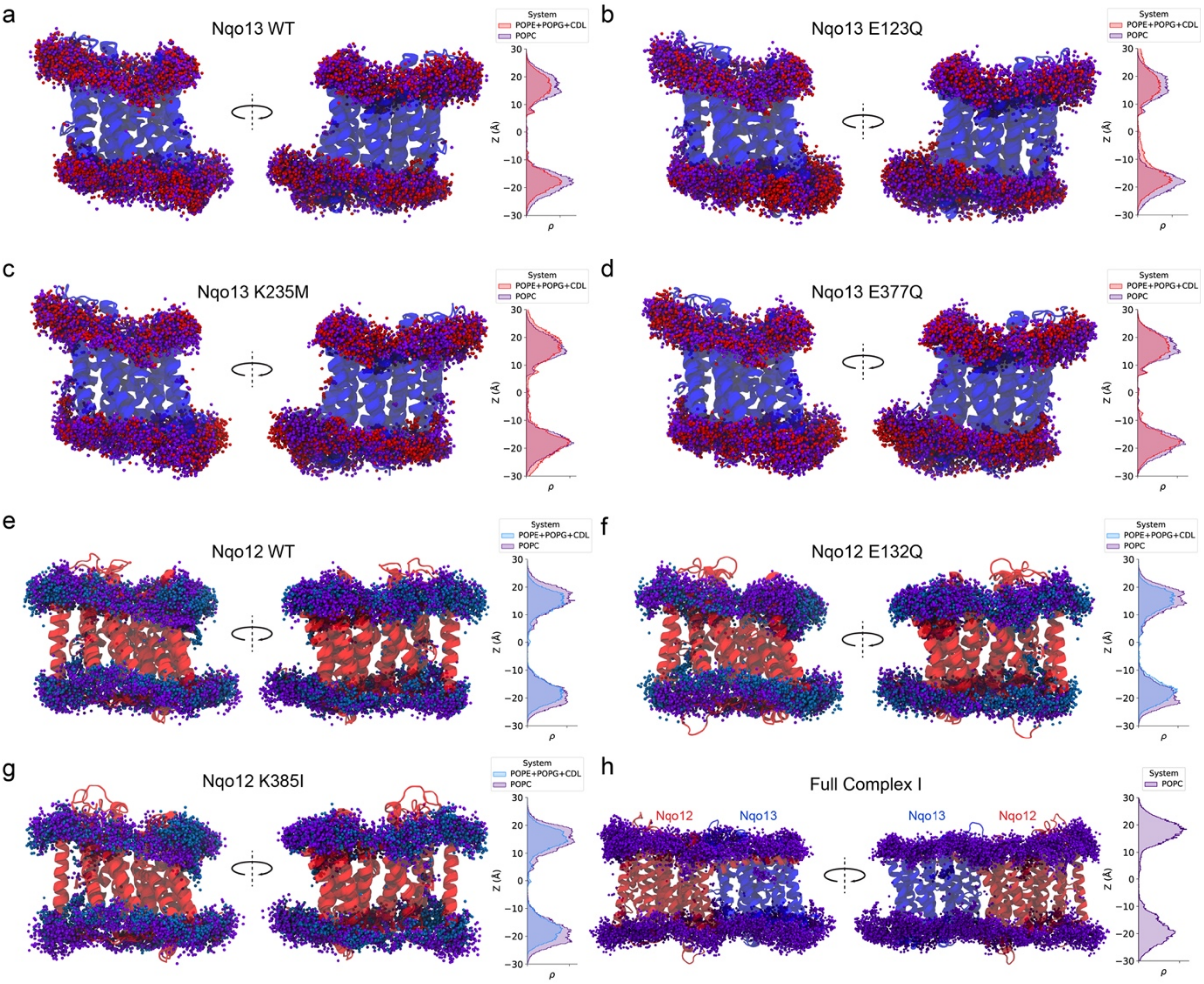

Supplementary Figure 19 | Hydration analysis of the protein-lipid interface in the isolated antiporter constructs and in the full Complex I. Left insets show a snapshot of the hydration ensemble with water molecules within 3 Å of protein and lipids. Right insets show the distribution of water molecules along the Z axis. The analyses were performed on the last 500 ns of the respective MD simulation, with structures extracted every 10 ns. **a-d**) MD simulations of the Nqo13 constructs. **a**) WT, **b**) E123Q, **c**) K235M, and **d**) E377Q. **e-g**) MD simulations of the Nqo12$^{\Delta TM}$ constructs. **e**) WT, **f**) E132Q, and **g**) K385I. **h**) MD simulations of the full Complex I, showing water molecules around the protein-lipid interface at the Nqo12/Nqo13 subunits (based on simulation data from Ref. 7). Data are provided in the Source Data file.

Supplementary Table 1 | Steady-state pH levels and kinetics of proton transfer based on proteoliposome experiments using a weak acid/base or by ATPase-driven proton transport.

	**Potassium acetate**			**Methylamine hydrochloride**		
**Construct**	**Plateau pH**	**SD**	***n***	**Plateau pH**	**SD**	***n***
EL	7.05	0.013	3	7.69	0.018	3
AqpZ	7.04	0.017	6	7.68	0.011	3
WT[12]	7.09	0.028	6	7.70	0.002	3
E132Q[12]	7.16	0.017	6	7.74	0.002	3
K385I[12]	7.05	0.013	6	7.63	0.003	3
WT[13]	7.11	0.007	6	7.65	0.008	3
E123Q[13]	7.14	0.002	3	7.67	0.004	3
K235M[13]	7.05	0.011	6	7.64	0.009	3
E377Q[13]	7.05	0.014	6	7.70	0.011	3

**ATP synthase**						
	**Linear fit**					
**Construct**	**avg. k_1**	**SD**	***n***	**Plateau ΔF^***	**SD**	***n***
EL	0.04	0.02	6	0.96	0.01	6
AqpZ	4.42	0.49	6	0.09	0.01	6
F_1F_o	5.56	0.40	6	0.07	0.01	6
WT[12]	2.53	0.28	6	0.33	0.04	6
E132Q[12]	0.58	0.18	6	0.71	0.06	6
K385I[12]	4.98	0.19	6	0.13	0.02	6
WT[13]	2.36	0.19	6	0.32	0.03	6
E123Q[13]	2.00	0.27	6	0.43	0.04	6
K235M[13]	2.72	0.28	5	0.31	0.03	5

*Extrapolated from a single exponential fit.

Supplementary Table 2 | Orientation of Nqo modules in proteoliposomes by NTA-Atto 647N labelling.

Construct	Orientation ratio (outward/inward)
**WT[12]**	31/69
**E132Q[12]**	18/82
**K385I[12]**	27/73
**WT[13]**	29/71
**E123Q[13]**	23/77
**K235M[13]**	21/79
**E377Q[13]**	28/72

Supplementary Table 3 | Predicted pK_as in the dissected antiporter-like subunit relative to the intact Complex I. The pK_a computed based on PDB ID:6I0D[6], and ΔpK_as of the isolated Nqo13 / Nqo12 constructs relative to WT intact CI based on MD ensemble (see *Supplementary methods*).

**Nqo13**	**pK_a** X-ray, CI full	**pK_a** X-ray, 13 only	**ΔpK_a** X-ray CI full - 13 only	**ΔpK_a** MD: CI full - WT/Nqo13	**ΔpK_a** MD: CI full - E123Q/Nqo13	**ΔΔpK_a** MD: WT - E123Q
**K282**	15.0	15.8	-0.8	-4.1	0.4	+4.4
**D228**	-4.7	-3.8	-0.9	-2.6	0.5	+3.1
**H218**	-0.7	0.5	-1.2	-4.8	2.2	+7.0
**K235**	8.6	9.8	-1.2	-2.4	-1.3	+1.1
**H292**	2.9	6.8	-3.9	-2.5	-1.9	+0.6
**E377**	5.8	4.7	+1.1	-5.3	-1.6	+3.7

**Nqo12**	**pK_a** X-ray, CI full	**pK_a** X-ray, 12 only	**ΔpK_a** X-ray CI full - 12 only	**ΔpK_a** MD: CI full - WT/Nqo12	**ΔpK_a** MD: CI full - E132Q/Nqo12	**ΔΔpK_a** MD: WT - E132Q
**E346**	4.4	4.6	-0.8	-0.1	+0.6	+0.7
**K292**	10.5	10.8	-0.3	+0.6	+0.4	-0.2
**H241**	-1.2	-0.6	-0.6	-3.8	-0.9	+2.9
**K329**	9.7	10.1	-0.4	-2.2	+1.8	+4
**H325**	-4.3	-4.1	-0.2	+0.0	+2.8	+2.8
**H321**	2.6	2.9	-0.3	+2.3	+3.4	+1.1
**K385**	13.5	13.7	-0.2	+4.7	+6.7	+2.0
**D386**	5.4	6.2	-0.8	-2.0	-0.4	+1.6

Supplementary Table 4 | List of MD simulations.

Simulation	System	Time (ns)	Modelled state	Dimensions (Å)	*N* atoms	*N* water molecules
S1	Nqo13-WT	1000	$Lys235^{+}/His292^{0}/Glu377^{-}$	83 x 81 x 89	59400	10900
S2	Nqo13-WT	1000	$Lys235^{+}/His292^{0}/Glu377^{-}$	83 x 81 x 89	59400	10900
S3	Nqo13-WT	1000	$Lys235^{0}/His292^{0}/Glu377^{0}$	83 x 81 x 89	59400	10900
S4	Nqo13-WT	1000	$Lys235^{0}/His292^{0}/Glu377^{0}$	83 x 81 x 89	59400	10900
S5	Nqo13-E123Q	1000	$Lys235^{+}/His292^{0}/Glu377^{-}$	83 x 81 x 89	59400	10900
S6	Nqo13-E123Q	1000	$Lys235^{+}/His292^{0}/Glu377^{-}$	83 x 81 x 89	59400	10900
S7	Nqo13-K235M	1000	$His292^{0}/Glu377^{-}$	83 x 81 x 89	59400	10900
S8	Nqo13-K235M	1000	$His292^{0}/Glu377^{-}$	83 x 81 x 89	59400	10900
S9	Nqo13-E377Q	1000	$Lys235^{+}/His292^{0}$	83 x 81 x 89	59400	10900
S10	Nqo13-E377Q	1000	$Lys235^{+}/His292^{0}$	83 x 81 x 89	59400	10900
S11	Nqo13-WT in PE:PG:CDL	500	$Lys235^{+}/His292^{0}/Glu377^{-}$	100 x 100 x 105	97900	19200
S12	Nqo13-WT in PE:PG:CDL	500	$Lys235^{+}/His292^{0}/Glu377^{-}$	100 x 100 x 105	97900	19200
S13	Nqo13-E123Q in PE:PG:CDL	500	$Lys235^{+}/His292^{0}/Glu377^{-}$	100 x 100 x 105	97900	19200
S14	Nqo13-E123Q in PE:PG:CDL	500	$Lys235^{+}/His292^{0}/Glu377^{-}$	100 x 100 x 105	97900	19200
S15	Nqo13-K235M in PE:PG:CDL	500	$His292^{0}/Glu377^{-}$	100 x 100 x 105	97900	19200
S16	Nqo13-K235M in PE:PG:CDL	500	$His292^{0}/Glu377^{-}$	100 x 100 x 105	97900	19200
S17	Nqo13-E377Q in PE:PG:CDL	500	$Lys235^{+}/His292^{0}$	100 x 100 x 105	97900	19200
S18	Nqo13-E377Q in PE:PG:CDL	500	$Lys235^{+}/His292^{0}$	100 x 100 x 105	97900	19200
S19	$Nqo12^{\Delta TH}$-WT	1000	$Lys329^{+}/Lys385^{+}$	101 x 103 x 95	98000	19400
S20	$Nqo12^{\Delta TH}$-WT	1000	$Lys329^{+}/Lys385^{+}$	101 x 103 x 95	98000	19400
S21	$Nqo12^{\Delta TH}$-E132Q	1000	$Lys329^{+}/Lys385^{+}$	101 x 103 x 95	98000	19400
S22	$Nqo12^{\Delta TH}$-E132Q	1000	$Lys329^{+}/Lys385^{+}$	101 x 103 x 95	98000	19400
S23	$Nqo12^{\Delta TH}$-K385I	1000	$Lys329^{+}$	101 x 103 x 95	98000	19400
S24	$Nqo12^{\Delta TH}$-K385I	1000	$Lys329^{+}$	101 x 103 x 95	98000	19400
S25	$Nqo12^{\Delta TH}$-WT in PE:PG:CDL	1000	$Lys329^{+}/Lys385^{+}$	109 x 107 x 109	125300	28200
S26	$Nqo12^{\Delta TH-}$WT in PE:PG:CDL	1000	$Lys329^{+}/Lys385^{+}$	109 x 107 x 109	125300	28200
S27	$Nqo12^{\Delta TH}$-E132Q in PE:PG:CDL	1000	$Lys329^{+}/Lys385^{+}$	109 x 107 x 109	125300	28200
S28	$Nqo12^{\Delta TH}$-E132Q in PE:PG:CDL	1000	$Lys329^{+}/Lys385^{+}$	109 x 107 x 109	125300	28200
S29	$Nqo12^{\Delta TH}$-K385I in PE:PG:CDL	1000	$Lys329^{+}$	109 x 107 x 109	125300	28200
S30	$Nqo12^{\Delta TH}$-K385I in PE:PG:CDL	1000	$Lys329^{+}$	109 x 107 x 109	125300	28200
**TOTAL**		**26 µs**				

Supplementary Table 5 | List of designed primers.

Oligonucleotide name	Sequence (5' → 3')
Nqo12$^{\Delta TH}$ extraction F	AACTTTAAGAAGGAGATATACCATGGCGCTTCTCGGGACGATTCTC
Nqo12$^{\Delta TH}$ extraction R	ACCTTGGAAGTATAAATTTTCCTTGCGCTGGAAGAAGACGAAACCCG
Nqo13 extraction F	ATGGTGGTGCTGGCGGTTC
Nqo13 extraction R	GCAGCAGAACCGCCAGCACCACCATGGTAT
Nqo12$^{\Delta TH}$ E132Q F	ATGTTCATCGGCTGGCAGGGGGTGGGCCTG
Nqo12$^{\Delta TH}$ E132Q R	CCAGCCGATGAACATCACC
Nqo13 E123Q F	TTTTCTTTCAGGCGGCGCTGATTCC
Nqo13 E123Q R	CCGCCTGAAAGAAAACGTAGAACACCAGCAGATCAC
Nqo13 K235M F	TGTACATGGTGGGTGTTTTTGCGTTCTTTC
Nqo13 K235M R	CACCCACCATGTACAGGGTGCCCAG
Nqo13 E377Q F	TCCGGGTCAATTCCTGACCCTGCTGG
Nqo13 E377Q R	AGGAATTGACCCGGAAAACCGCTC

Supplementary Table 6 | Sequences of studied Nqo constructs.

Construct name	Sequence
**Nqo12$^{\Delta TH}$ WT**	MALLGTILLPLLGFALLGLFGKRMREPLPGVLASGLVLASFLLGAGLLLSG<br>GARFQAEWLPGIPFSLLLDNLSGFMLLIVTGVGFLIHVYAIGYMGGDPGY<br>SRFFAYFNLFIAMMLTLVLADSYPVMFIGWEGVGLASFLLIGFWYKNAQY<br>ADSARKAFIVNRIGDLGFMLGMAILWALYGTLSISELKEAMEGPLKNPDLL<br>ALAGLLLFLGAVGKSAQIPLMVWLPDAMAGPTPVSALIHAATMVTAGVYL<br>IARSSFLYSVLPDVSYAIAVVGLLTAAYGALSAFGQTDIKKIVAYSTISQLG<br>YMFLAAGVGAYWVALFHVFTHAFFKALLFLASGSVIHALGGEQDVRKMG<br>GLWKHLPQTRWHALIGALALGGLPLLSGFWSKDAILAATLTYPFGGVGF<br>YVGALLVAVLTAMYAMRWFVLVFLGEERGHHHPHEAPPVMLWPNHLL<br>ALGSVLAGYLALPHPLPNVLEPFLKPALAEVEAHHLSLGAEWGLIALSAA<br>VALLGLWAGFVFFQRKGSAGSENLYFQGQFSKGEELFTGVVPILVELDG<br>DVNGHKFSVSGEGEGDATYGKLTLKFICTTGKLPVPWPTLVTTLTYGVQ<br>CFSRYPDHMKRHDFFKSAMPEGYVQERTISFKDDGNYKTRAEVKFEGD<br>TLVNRIELKGIDFKEDGNILGHKLEYNYNSHNVYITADKQKNGIKANFKIRH<br>NIEDGSVQLADHYQQNTPIGDGPVLLPDNHYLSTQSALSKDPNEKRDHM<br>VLLEFVTAAGITHGMDELYKTSHHHHHHHHHH*
**Nqo12$^{\Delta TH}$ E132Q**	MALLGTILLPLLGFALLGLFGKRMREPLPGVLASGLVLASFLLGAGLLLSG<br>GARFQAEWLPGIPFSLLLDNLSGFMLLIVTGVGFLIHVYAIGYMGGDPGY<br>SRFFAYFNLFIAMMLTLVLADSYPVMFIGW**Q**GVGLASFLLIGFWYKNAQY<br>ADSARKAFIVNRIGDLGFMLGMAILWALYGTLSISELKEAMEGPLKNPDLL<br>ALAGLLLFLGAVGKSAQIPLMVWLPDAMAGPTPVSALIHAATMVTAGVYL<br>IARSSFLYSVLPDVSYAIAVVGLLTAAYGALSAFGQTDIKKIVAYSTISQLG<br>YMFLAAGVGAYWVALFHVFTHAFFKALLFLASGSVIHALGGEQDVRKMG<br>GLWKHLPQTRWHALIGALALGGLPLLSGFWSKDAILAATLTYPFGGVGF<br>YVGALLVAVLTAMYAMRWFVLVFLGEERGHHHPHEAPPVMLWPNHLLA<br>LGSVLAGYLALPHPLPNVLEPFLKPALAEVEAHHLSLGAEWGLIALSAAV<br>ALLGLWAGFVFFQRKGSAGSENLYFQGQFSKGEELFTGVVPILVELDGD<br>VNGHKFSVSGEGEGDATYGKLTLKFICTTGKLPVPWPTLVTTLTYGVQCF<br>SRYPDHMKRHDFFKSAMPEGYVQERTISFKDDGNYKTRAEVKFEGDTLV<br>NRIELKGIDFKEDGNILGHKLEYNYNSHNVYITADKQKNGIKANFKIRHNIE<br>DGSVQLADHYQQNTPIGDGPVLLPDNHYLSTQSALSKDPNEKRDHMVLL<br>EFVTAAGITHGMDELYKTSHHHHHHHHHH*
**Nqo12$^{\Delta TH}$ K385I**	MALLGTILLPLLGFALLGLFGKRMREPLPGVLASGLVLASFLLGAGLLLSG<br>GARFQAEWLPGIPFSLLLDNLSGFMLLIVTGVGFLIHVYAIGYMGGDPGY<br>SRFFAYFNLFIAMMLTLVLADSYPVMFIGWEGVGLASFLLIGFWYKNAQY<br>ADSARKAFIVNRIGDLGFMLGMAILWALYGTLSISELKEAMEGPLKNPDLL<br>ALAGLLLFLGAVGKSAQIPLMVWLPDAMAGPTPVSALIHAATMVTAGVYL<br>IARSSFLYSVLPDVSYAIAVVGLLTAAYGALSAFGQTDIKKIVAYSTISQLG<br>YMFLAAGVGAYWVALFHVFTHAFFKALLFLASGSVIHALGGEQDVRKMG<br>GLWKHLPQTRWHALIGALALGGLPLLSGFWS**I**DAILAATLTYPFGGVGFY<br>VGALLVAVLTAMYAMRWFVLVFLGEERGHHHPHEAPPVMLWPNHLLAL<br>GSVLAGYLALPHPLPNVLEPFLKPALAEVEAHHLSLGAEWGLIALSAAVA<br>LLGLWAGFVFFQRKGSAGSENLYFQGQFSKGEELFTGVVPILVELDGDV<br>NGHKFSVSGEGEGDATYGKLTLKFICTTGKLPVPWPTLVTTLTYGVQCF<br>SRYPDHMKRHDFFKSAMPEGYVQERTISFKDDGNYKTRAEVKFEGDTL<br>VNRIELKGIDFKEDGNILGHKLEYNYNSHNVYITADKQKNGIKANFKIRHN<br>IEDGSVQLADHYQQNTPIGDGPVLLPDNHYLSTQSALSKDPNEKRDHMV<br>LLEFVTAAGITHGMDELYKTSHHHHHHHHHH*
**Nqo13 WT**	MVVLAVLLPVVFGALLLLGLPRALGVLGAGLSFLLNLYLFLTHPGGVAHA<br>FQAPLLPGAGVYWAFGLDGLSALFFLTIALTVFLGALVARVEGRFLGLAL<br>LMEGLLLGLFAARDLLVFYVFFEAALIPALLMLYLYGGEGRTRALYTFVLF<br>TLVGSLPMLAAVLGARLLSGSPTFLLEDLLAHPLQEEAAFWVFLGFALAF<br>AIKTPLFPLHAWLPPFHQENHPSGLADALGTLYKVGVFAFFRFAIPLAPEG<br>FAQAQGLLLFLAALSALYGAWVAFAAKDFKTLLAYAGLSHMGVAALGVFS<br>GTPEGAMGGLYLLAASGVYTGGLFLLAGRLYERTGTLEIGRYRGLAQSAP<br>GLAALALILFLAMVGLPGLSGFPGEFLTLLGAYKASPWLAALAFLSVIASAA<br>YALTAFQKTFWEEGGSGVKDLAGAEWGFALLSVLALLLMGVFPGYFARG<br>LHPLAEAFAKLLGGENLYFQGQFSKGEELFTGVVPILVELDGDVNGHKFS<br>VSGEGEGDATYGKLTLKFICTTGKLPVPWPTLVTTLTYGVQCFSRYPDH<br>MKRHDFFKSAMPEGYVQERTISFKDDGNYKTRAEVKFEGDTLVNRIELK<br>GIDFKEDGNILGHKLEYNYNSHNVYITADKQKNGIKANFKIRHNIEDGSVQ<br>LADHYQQNTPIGDGPVLLPDNHYLSTQSALSKDPNEKRDHMVLLEFVTA<br>AGITHGMDELYKTSHHHHHHHHHH*
**Nqo13 E123Q**	MVVLAVLLPVVFGALLLLGLPRALGVLGAGLSFLLNLYLFLTHPGGVAHAF

	QAPLLPGAGVYWAFGLDGLSALFFLTIALTVFLGALVARVEGRFLGLALLM<br>EGLLLGLFAARDLLVFYVFF**Q**AALIPALLMLYLYGGEGRTRALYTFVLFTLV<br>GSLPMLAAVLGARLLSGSPTFLLEDLLAHPLQEEAAFWVFLGFALAFAIKT<br>PLFPLHAWLPPFHQENHPSGLADALGTLYKVGVFAFFRFAIPLAPEGFAQ<br>AQGLLLFLAALSALYGAWVAFAAKDFKTLLAYAGLSHMGVAALGVFSGTP<br>EGAMGGLYLLAASGVYTGGLFLLAGRLYERTGTLEIGRYRGLAQSAPGLA<br>ALALILFLAMVGLPGLSGFPGEFLTLLGAYKASPWLAALAFLSVIASAAYAL<br>TAFQKTFWEEGGSGVKDLAGAEWGFALLSVLALLLMGVFPGYFARGLHP<br>LAEAFAKLLGGENLYFQGQFSKGEELFTGVVPILVELDGDVNGHKFSVSG<br>EGEGDATYGKLTLKFICTTGKLPVPWPTLVTTLTYGVQCFSRYPDHMKRH<br>DFFKSAMPEGYVQERTISFKDDGNYKTRAEVKFEGDTLVNRIELKGIDFKE<br>DGNILGHKLEYNYNSHNVYITADKQKNGIKANFKIRHNIEDGSVQLADHYQ<br>QNTPIGDGPVLLPDNHYLSTQSALSKDPNEKRDHMVLLEFVTAAGITHGM<br>DELYKTSHHHHHHHHHH*
**Nqo13 K235M**	MVVLAVLLPVVFGALLLLGLPRALGVLGAGLSFLLNLYLFLTHPGGVAHAF<br>QAPLLPGAGVYWAFGLDGLSALFFLTIALTVFLGALVARVEGRFLGLALLM<br>EGLLLGLFAARDLLVFYVFFEAALIPALLMLYLYGGEGRTRALYTFVLFTLV<br>GSLPMLAAVLGARLLSGSPTFLLEDLLAHPLQEEAAFWVFLGFALAFAIKT<br>PLFPLHAWLPPFHQENHPSGLADALGTLY**M**VGVFAFFRFAIPLAPEGFAQ<br>AQGLLLFLAALSALYGAWVAFAAKDFKTLLAYAGLSHMGVAALGVFSGTP<br>EGAMGGLYLLAASGVYTGGLFLLAGRLYERTGTLEIGRYRGLAQSAPGLA<br>ALALILFLAMVGLPGLSGFPGEFLTLLGAYKASPWLAALAFLSVIASAAYAL<br>TAFQKTFWEEGGSGVKDLAGAEWGFALLSVLALLLMGVFPGYFARGLHP<br>LAEAFAKLLGGENLYFQGQFSKGEELFTGVVPILVELDGDVNGHKFSVSG<br>EGEGDATYGKLTLKFICTTGKLPVPWPTLVTTLTYGVQCFSRYPDHMKRH<br>DFFKSAMPEGYVQERTISFKDDGNYKTRAEVKFEGDTLVNRIELKGIDFKE<br>DGNILGHKLEYNYNSHNVYITADKQKNGIKANFKIRHNIEDGSVQLADHYQ<br>QNTPIGDGPVLLPDNHYLSTQSALSKDPNEKRDHMVLLEFVTAAGITHGM<br>DELYKTSHHHHHHHHHH*
**Nqo13 E377Q**	MVVLAVLLPVVFGALLLLGLPRALGVLGAGLSFLLNLYLFLTHPGGVAHAF<br>QAPLLPGAGVYWAFGLDGLSALFFLTIALTVFLGALVARVEGRFLGLALLM<br>EGLLLGLFAARDLLVFYVFFEAALIPALLMLYLYGGEGRTRALYTFVLFTLV<br>GSLPMLAAVLGARLLSGSPTFLLEDLLAHPLQEEAAFWVFLGFALAFAIKT<br>PLFPLHAWLPPFHQENHPSGLADALGTLYKVGVFAFFRFAIPLAPEGFAQ<br>AQGLLLFLAALSALYGAWVAFAAKDFKTLLAYAGLSHMGVAALGVFSGTP<br>EGAMGGLYLLAASGVYTGGLFLLAGRLYERTGTLEIGRYRGLAQSAPGLA<br>ALALILFLAMVGLPGLSGFPG**Q**FLTLLGAYKASPWLAALAFLSVIASAAYAL<br>TAFQKTFWEEGGSGVKDLAGAEWGFALLSVLALLLMGVFPGYFARGLH<br>PLAEAFAKLLGGENLYFQGQFSKGEELFTGVVPILVELDGDVNGHKFSVS<br>GEGEGDATYGKLTLKFICTTGKLPVPWPTLVTTLTYGVQCFSRYPDHMK<br>RHDFFKSAMPEGYVQERTISFKDDGNYKTRAEVKFEGDTLVNRIELKGI<br>DFKEDGNILGHKLEYNYNSHNVYITADKQKNGIKANFKIRHNIEDGSVQL<br>ADHYQQNTPIGDGPVLLPDNHYLSTQSALSKDPNEKRDHMVLLEFVTA<br>AGITHGMDELYKTSHHHHHHHHHH*

Supplementary Table 7 | Expression strains and tested plasmids.

Expression strain	Plasmid	Construct	Properties
BL21 (DE3), C41 (DE3), C43 (DE3)	pET21a	His-TEV-Nqo13	No expression
BL21 (DE3), C41 (DE3), C43 (DE3)	pBAD	His-TEV-Nqo13	No expression
BL21 (DE3), C41 (DE3), C43 (DE3)	pWALDO	Nqo13-TEV-GFP-His	Low expression
BL21 (DE3)	pWALDO	Nqo12-TEV-sfGFP-His	Low expression, no binding to Ni-NTA
LEMO21 (DE3)	pWALDO	Nqo12-linker-TEV-sfGFP-His	Successful
LEMO21 (DE3)	pWALDO	Nqo13-TEV-His	Successful

Supplementary Table 8 | Benchmarking the proton transfer energetics. The model system contains N=137 atoms (see also Supplementary Fig. 9). Electronic energies were evaluated at def2-TZVP/ε=4 (DFT)[17-23] and def2-TZVPPD/ε=4 (RPA)[24] levels.

State	B3LYP-D3	CAM-B3LYP-D3	CAMh-B3LYP-D3	TPSSh-D3	ωB97X-D	RPA
**I**	0.0	0.0	0.0	0.0	0.0	0.0
**II**	6.3	6.1	6.4	6.1	6.9	8.1
**III**	4.6	4.5	4.4	4.7	4.7	4.8
**IV**	17.8	17.6	17.8	16.4	19.1	18.8
**V**	8.3	8.7	8.4	8.4	8.0	6.5

Supplementary Table 9 | List of QM/MM simulations

Simulation	System	QM region	Description	Length (ps)	Method
1	Nqo13 WT	K235, H292, E377, T232, S291, S318, Y321, S402, Y405, 10 H_2O (N=137)	Lateral proton transfer pathway from K235 to E377	75 x 2.3 ps = 173 ps	US/WHAM
2	Nqo13 E123Q	K235, H292, E377, T232, S291, S318, Y321, S402, Y405, 10 H_2O (N=137)	Lateral proton transfer pathway from K235 to E377	75 x 2.3 ps = 173 ps	US/WHAM
3	Nqo12 WT	K329, K385, H321, H325, S298, Q302, Y305, F328, S384, T413, Y416, 4 H_2O (N=149)	Lateral proton transfer pathway from K329 to K385	52 x 2.3 ps = 120 ps	US/WHAM
4	Nqo12 E132Q	K329, K385, H321, H325, S298, Q302, Y305, F328, S384, T413, Y416, 4 H_2O (N=149)	Lateral proton transfer pathway from K329 to K385	52 x 2.3 ps = 120 ps	US/WHAM
5	Nqo13 WT	K235, H211, T232, T322, F217, H218, D228, F326, N221, K282, 9 H_2O (N=148)	N-side medial proton transfer from K282 to K235	10 x 22.5 ps = 225 ps	MWE/MBAR

Supplementary Methods

Derivation of an analytical model for ion-pair induced barrier tuning

An analytical model of the ion-pair induced barrier reduction was derived based on an electrostatic consideration. To this end, the influence of the ion-pair opening on the transition state energy was modelled by considering the electrical tuning effect of moving the proton from the middle lysine residue (Lys235 in Nqo13) to a nearby protonated water species (H_3O^+ or $H_5O_2^+$ at the transition state $E^‡$), located at a distance *a* from the latter (Supplementary Fig. 7). The effect of the electrostatic tuning was modelled by summing over all charged interactions,

$$V_{ij}(r) = \frac{e^2}{4\pi\epsilon\epsilon_0}\sum_{i>j}\frac{q_i q_j}{r_{ij}} \quad (1)$$

leading to the barrier tuning based on ion-pair distance *x*,

$$\Delta E^‡(x) = \alpha\left[\frac{1}{R+a-(x-d)} - \frac{1}{R+d+a} - \frac{1}{R-(x-d)} + \frac{1}{R+d}\right] \quad (2)$$

where $\alpha = q_i q_j e^2/4\pi\varepsilon\varepsilon_0$ = 332 kcal mol^{-1} Å^{-1}/ε, *R* is the distance between the middle lysine and the ion pair, *d* and *x* are the distances between the ion-paired residues in the respective closed and open conformations, and, *a* is the distance between the protonated water species and Lys235. The dielectric constant of the medium was modelled with ε =10. The geometry of the model is shown in Supplementary Fig. 7, with *R*= 15.9 Å, *a* = 9.2 Å, *d* = 3.2 Å.

Poisson-Boltzmann Electrostatic with Monte Carlo sampling

pK_a values for titratable residues in Nqo12 and Nqo13 were estimated based on Poisson-Boltzmann (PB) electrostatic calculations together with Monte Carlo (MC) sampling. To this end, the protein residues were treated as explicit atoms using the CHARMM36m force field[1]. The protein surroundings were modelled as an inhomogeneous polarisable low-dielectric (ε=10) medium, with explicit lipid molecules included from the MD simulations. The bulk water was modelled as a homogenous high dielectric medium (ε=80). The PB equations were solved numerically using the Adaptive Poisson–Boltzmann Solver (APBS)[2]. The 2^N protonation states in the protein were sampled by allowing for single, double, and triple moves in the MC routine, as implemented in Karlsberg+[3-5]. The sidechain atoms were optimised upon protonation state changes using the ABNR minimiser in CHARMM[25]. The pK_as were estimated based on their shifts from experimental solvent (water) pK_a values,

pK_a (protein) = pK_a (water) + ΔpK_a (water → protein)

with solvent pK_as of Glu (4.1), Asp (3.9), Tyr (10.1), His (6.04), and Lys (10.5). The ΔpK_a term was calculated based on changes in the electrostatic interactions of the protonated (AH) and deprotonated (A) species with surrounding protein background charges (ΔG_{back}), the Born desolvation energy (ΔG_{Born}) for moving AH and A from water (ε=80) to the protein medium (ε=10), and from interaction of the 2^N possible protonation states in the protein medium ($\Delta G_{ij}^{protein}$),

$$\Delta pK_a \text{ (water} \rightarrow \text{protein)} = \Delta\Delta G^{water\rightarrow protein} / 2.303\ RT \quad (3)$$

with

$$\Delta\Delta G^{water\rightarrow protein} = \Delta G_{Born} + \Delta G_{back} + \sum_{i\neq j} \Delta G_{ij}^{protein} \quad (4)$$

The calculations were performed for the isolated Nqo12 and Nqo13 models based on the X-ray structure of *Thermus thermophilus* Complex I (PDB ID: 6i0d[6]), and based on MD simulations of the WT-Nqo13, E132Q-Nqo13, WT-Nqo12, E123Q-Nqo12 models (see *Methods*, main text). The pK_a shifts were also compared to MD simulations of the intact Complex I from *T. thermophilus*[7].

Analysis of hydration profiles

The analysis and visualisation of hydration profiles from MD simulations were carried out using CAVER v.3.0[8] and Visual Molecular Dynamics (VMD)[9]. Proton pathways were first characterised by starting the tunnel search from Lys329 in Nqo12$^{\Delta TH}$ or Lys235 in Nqo13, using a probe radius set to 0.9 Å. Sidechains of histidine residues, Phe378^{Nqo13}, and the headgroups of charged residues (Glu, Asp, Lys, Arg) were excluded from the initial tunnel search. The hydration profiles were computed based on the identified tunnels, by probing the water occupancy within a 2 Å radius along the tunnel, based on the last 100 ns of a given trajectory. The hydration percentage was calculated as the ratio between occupied water molecules at a given tunnel coordinate and the total number of analysed frames.

Constant-pH MD simulations

Constant-pH MD (cphmd) simulations were performed based on the 1 μs MD simulations of Nqo13 embedded in a POPC membrane and solvated in a water box (see main text *Methods*). The cphmd simulations were performed using 15 independent simulations, with 20 replicas each, sampling a pH range from 0.5 to 13.5, and with initial protonation states assigned from the PBE/MC calculations (see above). Each simulation included 1000 minimisation steps, followed by 500 cycles of 1000-10000 production steps and 7500 non-equilibrium switching steps, leading to a total simulation length of *ca*. 50 ns. Final pK_a values were computed based on all sampled conformations using the PBE/MC methodology (see above) by re-weighing each conformation by its protonation probability and electrostatic energy to derive average protonation states. The cphmd simulations were performed, as implemented in NAMD[10-12], and pK_a calculations using an in-house APBS/Karlsberg+ implementation[2-5].

Supplementary Discussion

Conformational changes coupled to proton transport

To test how the proton transfer reactions coupled to conformational changes in the antiporter-like subunits, we performed additional MD simulations in multiple states. In the WT-Nqo13, we find that upon transfer of the proton from Lys235 to Glu377, mimicking a proton transfer step along the central pathway, the sidechain of His292 flips to a different rotameric state, suggesting that the conformation of His292 could affect the back transfer of protons in the reverse direction (*cf.* also Refs.[13, 14], Supplementary Fig. 1g). Moreover, we note that the deprotonation of the Lys235 leads conformational changes in conserved residues in TM7a/b and TM8 (Supplementary Fig. 1h) that could regulate the proton uptake from the N-side bulk. The constant pH-MD simulations (see *Supplementary Methods*) further support conformational changes around the broken TM7a/b helix, suggesting that the effective pK_a of Lys235 is around 9.3 in the WT-Nqo13, and shifts to 6.5 for E132Q-Nqo13 (Supplementary Fig. 16).

Supplementary References

1. Best, R. B., Zhu, X., Shim, J., Lopes, P. E. M.;,Mittal, J., Feig, M. & MacKerell, A. D. Optimization of the additive CHARMM all-atom protein force field targeting improved sampling of the backbone φ, ψ and side-chain χ_1 and χ_2 Dihedral Angles. *J. Chem. Theory Comput.* **8**, 3257– 3273 (2012)
2. Baker, N. A., Sept, D., Joseph, S., Holst M. J. & McCammon, J. A. Electrostatics of nanosystems: Application to microtubules and the ribosome. *Proc. Natl. Acad. Sci. USA* **98**, 10037–10041 (2001).
3. Meyer, T., Kieseritzky, G., & Knapp, E.-W. Electrostatic pKa computations in proteins: Role of internal cavities. *Proteins* **79**, 3320-3332 (2011).
4. Kieseritzky, G., & Knapp, E.-W. Improved pKa prediction: combining empirical and semimicroscopic methods. *J. Comput. Chem.* **29**, 2575-2581 (2008).
5. Rabenstein, B., Ullmann, G. M., & Knapp, E.-W Calculation of protonation patterns in proteins with structural relaxation and molecular ensembles - application to the photosynthetic reaction center. *Eur. Biophys. J.* **27**, 626-637 (1998).
6. Gutiérrez-Fernández, J., Kaszuba, K., Minhas, G. S., Baradaran, R., Tambalo, M., Gallagher, D. T. & Sazanov, L. A. Key role of quinone in the mechanism of respiratory complex I. *Nat. Commun.* **11**, 4135 (2020).
7. Di Luca, A., Gamiz-Hernandez A. P. & Kaila, V. R. I., Symmetry-related proton transfer pathways in respiratory complex I. *Proc. Nat. Acad. Sci. U S A* **114**, E6314-E6321 (2017).
8. Chovancova, E., *et al.* CAVER 3.0: a tool for the analysis of transport pathways in dynamic protein structures. *PLoS Comput. Biol.,* **8**, e1002708 (2012).
9. Humphrey, W. A., Dalke, A. & Schulten, K. VMD: Visual molecular dynamics. *J. Mol. Graph.* **14**, 33–38 (1996).
10. Stern, H.A. Molecular simulation with variable protonation states at constant pH. *J. Chem. Phys.* 126 (2007).
11. Chen, Y. & Roux, B. Constant-pH Hybrid Nonequilibrium Molecular Dynamics–Monte Carlo Simulation Method. *J. Chem. Comput. Theo.* **11**, 3919-3931 (2015).
12. Radak, B.K. *et al.* Constant-pH Molecular Dynamics Simulations for Large Biomolecular Systems. *J. Chem. Comput. Theo.* **13,** 5933-5944 (2017).
13. Mühlbauer, M. E. *et al.*, Water-gated proton transfer dynamics in respiratory complex I. *J. Am. Chem. Soc.* **142**, 13718–13728 (2020).
14. Lee, Y., Haapanen, O., Altmeyer, A., Kühlbrandt, W., Sharma, V. & Zickermann, V. Ion transfer mechanisms in Mrp-type antiporters from high resolution cryoEM and molecular dynamics simulations. *Nat. Commun.* **13**, 6091. (2022).
15. Scarciglia A, Di Gregorio E, Aime S, Ferrauto G. Effects of Cations on HPTS Fluorescence and Quantification of Free Gadolinium Ions in Solution; Assessment of Intracellular Release of Gd^{3+} from Gd-Based MRI Contrast Agents. *Molecules*. **27**, 2490 (2022).
16. Sievers, F., Wilm, A., Dineen, D.; Gibson, T. J., Karplus, K., Li, W., Lopez, R., McWilliam, H., Remmert, M., Söding, J., Thompson, J. D. & Higgins, D. G. Fast, Scalable Generation of High-quality Protein Multiple Sequence Alignments Using Clustal Omega. *Mol. Syst. Biol.* **7,** 539, (2011).
17. Becke, A. D. Density-functional Thermochemistry. III. The Role of Exact Exchange. *J. Chem. Phys.* **98,** 5648–5652 (1993).
18. Lee, C., Yang, W. & Parr, R. G. Development of the Colle-Salvetti Correlation-Energy Formula into a Functional of the Electron Density. *Phys. Rev. B* **37**, 785–789 (1988).
19. Grimme, S., Antony, J., Ehrlich, S. & Krieg, H. A Consistent and Accurate *Ab Initio* Parametrization of Density Functional Dispersion Correction (DFT-D) for the 94 Elements H-Pu. *J. Chem. Phys.* **132**, 154104 (2010).
20. Yanai, T., Tew, D. P. & Handy, N. C. A New Hybrid Exchange–Correlation Functional Using the Coulomb-Attenuating Method (CAM-B3LYP). *Chemical Physics Letters* **393**, 51–57 (2004).
21. Shao, Y., Mei, Y., Sundholm, D. & Kaila, V. R. I. Benchmarking the Performance of Time-Dependent Density Functional Theory Methods on Biochromophores. *J. Chem. Theory Comput.* **16**, 587–600 (2020).
22. Tao, J., Perdew, J. P., Staroverov, V. N. & Scuseria, G. E. Climbing the Density Functional Ladder: Nonempirical Meta–Generalized Gradient Approximation Designed for Molecules and Solids. *Phys. Rev. Lett.* **91**, 146401 (2003).
23. Chai, J.-D. & Head-Gordon, M. Long-Range Corrected Hybrid Density Functionals with Damped Atom–Atom Dispersion Corrections. *Phys. Chem. Chem. Phys.* **10**, 6615 (2008).
24. Chen, G.P., Voora, V.K., Agee, M.M., Balasubramani, S.G. & Furche, F. Random-Phase Approximation Methods. *Annu. Rev. Phys. Chem.* **68**, 421–445 (2017).
25. Brooks, B.R. *et al.* CHARMM: The biomolecular simulation program. *J. Comput. Chem.* **30**, 1545-1614 (2009).
